# An RXLR effector PlAvh142 from *Peronophythora litchii* triggers plant cell death and contributes to virulence

**DOI:** 10.1111/mpp.12905

**Published:** 2020-01-07

**Authors:** Junjian Situ, Liqun Jiang, Xiaoning Fan, Wensheng Yang, Wen Li, Pinggen Xi, Yizhen Deng, Guanghui Kong, Zide Jiang

**Affiliations:** ^1^ Department of Plant Pathology/Guangdong Province Key Laboratory of Microbial Signals and Disease Control South China Agricultural University Guangzhou China; ^2^ Guangdong Province Key Laboratory of New Technology in Rice Breeding/Rice Research Institute Guangdong Academy of Agricultural Sciences Guangzhou China

**Keywords:** cell death, *Peronophythora litchii*, plant immunity, RXLR effector

## Abstract

Litchi downy blight, caused by the phytopathogenic oomycete *Peronophythora litchii*, results in tremendous economic loss in litchi production every year*.* To successfully colonize the host cell, *Phytophthora* species secret hundreds of RXLR effectors that interfere with plant immunity and facilitate the infection process. Previous work has already predicted 245 candidate RXLR effector‐encoding genes in *P. litchii*, 212 of which have been cloned and tested for plant cell death‐inducing activity in this study. We found three such RXLR effectors could trigger plant cell death through transient expression in *Nicotiana benthamiana*. Further experiments demonstrated that PlAvh142 could induce cell death and immune responses in several plants. We also found that PlAvh142 localized in both the cytoplasm and nucleus of plant cells. The cytoplasmic localization was critical for its cell death‐inducing activity. Moreover, deletion either of the two internal repeats in PlAvh142 abolished the cell death‐inducing activity. Virus‐induced gene silencing assays showed that cell death triggered by PlAvh142 was dependent on the plant transduction components *RAR1* (require for *Mla12* resistance), *SGT1* (suppressor of the G2 allele of *skp1*) and *HSP90* (heat shock protein 90). Finally, knockout of *PlAvh142* resulted in significantly attenuated *P. litchii* virulence on litchi plants, whereas the *PlAvh142‐*overexpressed mutants were more aggressive. These data indicated that PlAvh142 could be recognized in plant cytoplasm and is an important virulence RXLR effector of *P. litchii*.

## Graphical abstract

The RXLR effector PlAvh142 from *Peronophythora litchii* triggers cell death, which is dependent on plant *RAR1*, *SGT1,* and *HSP90*. Knockout of *PlAvh142* by CRISPR/Cas9 attenuates the pathogenicity of *P. litchii*.

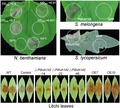

## 1 INTRODUCTION

In the arms race between plants and microbial plant pathogens, plants develop complex and multilayered immune systems for self‐defence: pathogen‐ or microbe‐associated molecular patterns (PAMPs/MAMPs)‐triggered immunity (PTI), mediated by pattern recognition receptors (PRRs) and effector‐triggered immunity (ETI), mediated by the specific disease resistance (R) proteins that recognize avirulence (AVR) effectors (Chisholm *et al.*, 2006; Jones and Dangl, 2006; Dodds and Rathjen, 2010; Boutrot and Zipfel, 2017; Cesari, 2018; Han, 2018). In plants, perception of PAMPs or effectors activates a complicated signal transduction network, including mitogen‐activated protein kinase (MAPK) cascades, and/or chemical signalling by plant hormones and transcriptional regulation via transcription factors (Pitzschke *et al.*, 2009; Wang *et al*., 2019b). The immune activation culminates in a series of physiological changes in the plant, such as reactive oxygen species (ROS) production, cell wall fortification, and the localized rapid cell death known as the hypersensitive response (HR) (Ingle *et al.*, 2006; Franceschetti *et al.*, 2017).

Oomycetes are a group of straminipilous organisms that are phylogenetically distant from true fungi (Göker *et al.*, 2007; Beakes *et al.*, 2012). Notably, phytopathogenic oomycetes are a constant threat to many important crops, rendering enormous crop yield losses globally (Tyler, 2007; Lamour *et al.*, 2012; Fry *et al.*, 2015). The phytopathogenic oomycetes could use the complement effectors, secreted into either apoplastic or cytoplasmic regions, as major virulence factors for successful infection and causing disease symptoms (Tyler *et al.*, 2006; Wang and Wang, 2018a). Among them, the cytoplasmic RXLR effectors that contain an N‐terminal signal peptide followed by the conserved Arg‐any amino acid‐Leu‐Arg (RXLR) motif, are a large superfamily of virulence proteins in oomycetes (Rehmany *et al.*, 2005; Whisson *et al.*, 2007). The RXLR motif is located within 30 residues downstream of the secretion signal cleavage site and is frequently followed by a less conserved Asp‐Glu‐Glu‐Arg (dEER) motif (Wawra *et al.*, 2012). It is suggested that the RXLR‐dEER motif is involved in translocating effector proteins from haustoria into host cells (Dou *et al.*, 2008; Kale *et al.*, 2010). RXLR effectors have become a focus for studying plant–pathogen interaction in the past decade, with numerous effector genes identified and characterized in *Phytophthora* and downy mildew species (Tyler *et al.*, 2006; Haas *et al.*, 2009; Baxter *et al.*, 2010; Yin *et al.*, 2017). Many studies have reported that RXLR effectors are involved in the suppression of PTI and/or ETI (Wang *et al.*, 2011; Kong *et al.*, 2017; Fan *et al.*, 2018) Additionally, some of them can trigger immune response‐related cell death, for example *Phytophthora sojae* Avh238 (Yang *et al.*, 2017), *Phytophthora capsici* Avh1 (Chen *et al.*, 2019), and *Plasmopara viticola* RXLR16 (Xiang *et al.*, 2017).

After being secreted by oomycetous pathogens, the RXLR effectors are transported to a range of subcellular localizations in plant cells, including the nucleus, cytoplasm, or plasma membrane, which often associates with their functions and/or mode of action (McLellan *et al.*, 2013; Xiang *et al.*, 2016). The study of subcellular localization of RXLR effectors from *Phytophthora infestans* revealed that the nucleocytoplasmic distribution in plant cells is the most common pattern (Wang *et al*., 2019a). *P*. *sojae* Avh238 triggers cell death when it is present in the nucleus (Yang *et al.*, 2017). In addition, plasma membrane localization of *P. sojae* Avh241 is required for its cell death‐inducing activity (Yu *et al.*, 2012).

*Peronophythora litchii* is one of the most destructive oomycete pathogens, causing downy blight on litchi fruits as well as tender leaves and panicles rot of litchi plants (Zheng *et al.*, 2019). As a hemibiotrophic pathogen, *P. litchii* undergoes biotrophic and necrotrophic phases during infection. However, fewer studies have been conducted on the functions of *P. litchii* genes, hence there is scarcity of information about its pathogenesis and the litchi–*P*. *litchii* interaction mechanisms (Jiang *et al.*, 2017, 2018; Kong *et al*., 2019). The identification and/or investigation of RXLR effectors in *P. litchii* lags behind that for other *Phytophthora* and downy mildew species, with only bioinformatics prediction of 245 putative RXLR effector genes (Ye *et al.*, 2016). Therefore, exploring the roles of *P. litchii* RXLR effectors in host–pathogen interaction could potentially reveal mechanisms underlying oomycete pathogenicity and host resistance, which would be beneficial for developing disease control strategies.

In this study, systematic screening identified three *P. litchii* RXLR effectors, PlAvh23, PlAvh133, and PlAvh142, that are able to induce cell death by transient expression in *Nicotiana benthamiana*. Further experiments showed that PlAvh142 could induce cell death in different plants, therefore we focused on the investigation of PlAvh142 functions. We found that the internal repeats are indispensable for the cell death‐inducing activity. PlAvh142 could localize in both cytoplasm and nucleus in the plant cell, but its cytoplasmic localization was demonstrated to be essential for triggering cell death. Through virus‐induced gene silencing (VIGS) assays, we found that cell death triggered by PlAvh142 is dependent on *RAR1*, *SGT1,* and *HSP90*, which suggests that PlAvh142 might be perceived by the innate immune system in plant. Finally, by genetic manipulation we proved that *PlAvh142* is important for *P. litchii* infection to its host plant litchi. The work provides a critical foundation for further dissection of the roles of *P. litchii* RXLR effectors in interaction with plant immunity.

## 2 RESULTS

### 2.1 PlAvh142 can induce cell death in plants

**Figure 1:**
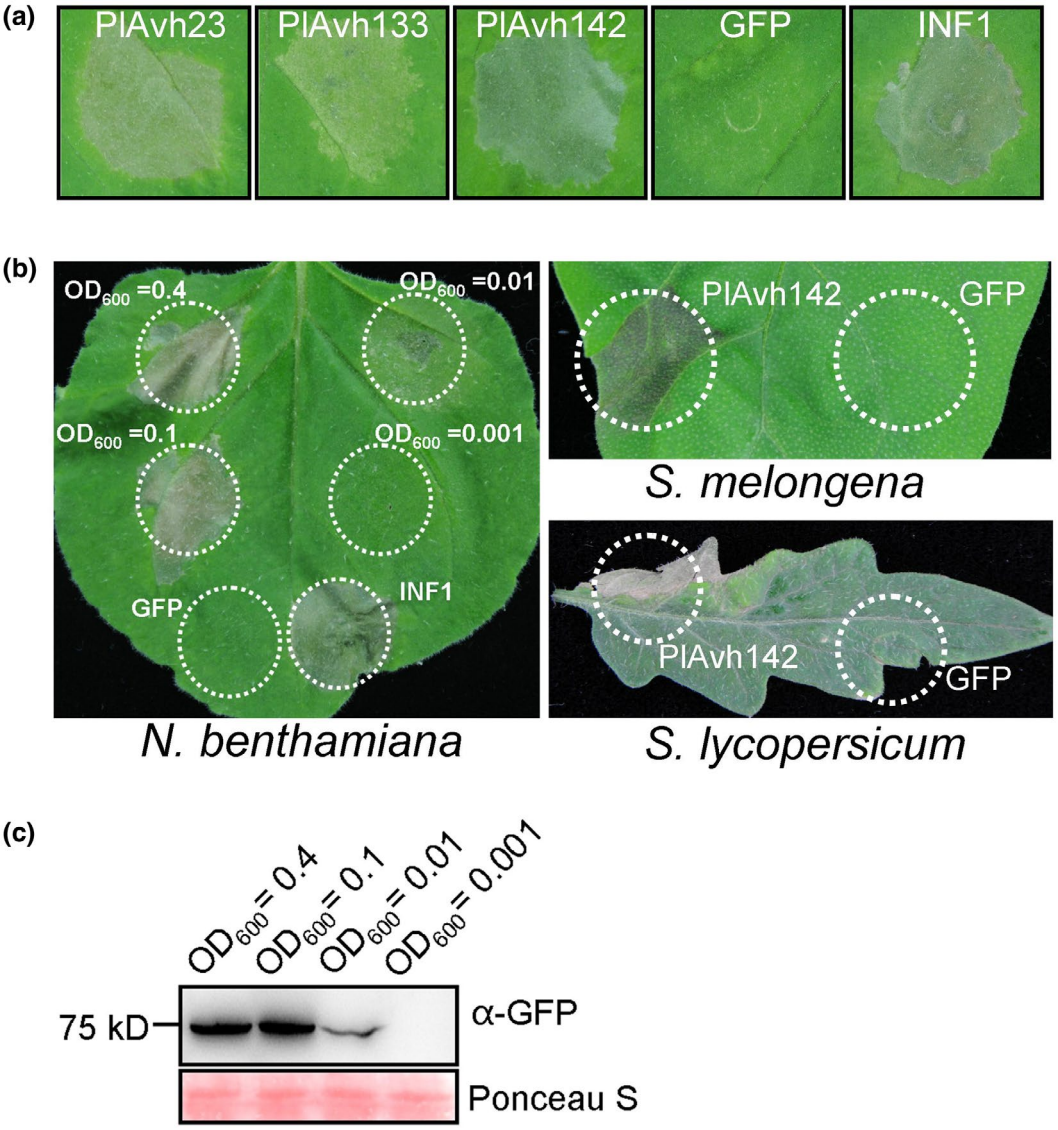
Analysis of the cell death phenotype of RXLR effectors from *Peronophythora litchii*. (a) Cell death triggered by *P. litchii* RXLR effectors in *Nicotiana benthamiana* leaves. Leaves of *N. benthamiana* were infiltrated with *Agrobacterium tumefaciens* carrying pBIN::*GFP‐PlAvh142*. Photographs were taken at 5 days post‐agroinfiltration (dpa). Green fluorescent protein (GFP) and INF1 were used as negative and positive control, respectively. (b) Cell death symptoms induced by PlAvh142 in different plants. *A. tumefaciens* carrying PlAvh142 was infiltrated into the leaves of *N. benthamiana*, *Solanum melongena*, and *Solanum lycopersicum*. Photographs were taken at 5 dpa. GFP was used as negative control. (c) Confirmation of proteins accumulation. Total proteins were extracted from *N. benthamiana* leaves at 2 dpa. Immunoblot analyses were performed using anti‐GFP (top panel) antibody. Ponceau S staining of total protein serves as loading control (bottom panel). Representative images were chosen for the results obtained from three independent experiments

To systematically investigate the functions of *P. litchii* RXLR effectors, 212 out of 245 predicted RXLR effectors (Ye *et al.*, 2016) were successfully cloned and then transiently expressed individually in *N. benthamiana* to test their cell death‐inducing activity. Effector gene cloning and cell‐death induction analysis are summarized in Table S1. Three RXLR effectors, PlAvh23, PlAvh133, and PlAvh142, were found to be able to induce cell death at 3–7 days post‐agroinfiltration (dpa) (Figure 1a). Among them, PlAvh142 exhibited strong cell death‐inducing activity in *N. benthamiana*, *Solanum melongena,* and *Solanum lycopersicum* (Figure 1b). Thus, this effector was selected for further analyses in this study. The cell death activity was further tested by infiltrating *Agrobacterium tumefaciens* (carrying a green fluorescent protein [GFP]‐tagged PlAvh142) with OD_600_ = 0.001, 0.01, 0.1, and 0.4, respectively, in *N. benthamiana* leaves (Figure 1b). The results showed that PlAvh142 induced cell death with OD_600_ = 0.01, 0.1 or 0.4. Western blot assays confirmed the expression of GFP‐PlAvh142 in all the agroinfiltrated leaves except for the OD_600_ of 0.001 (Figure 1c). Sequence analysis indicated that *PlAvh142* encodes a protein of 466 amino acids with a signal peptide from 1 to 20 amino acids. Moreover, it harbours the typical N‐terminal RXLR‐dEER motif (50–71 amino acids) and a potential unknown functional C‐terminus (Figure S1). Overall, we identified RXLR effectors from *P. litchii* that could induce plant cell death.

### 2.2 Expression of PlAvh142 activates various immune responses in *N. benthamiana*

**Figure 2:**
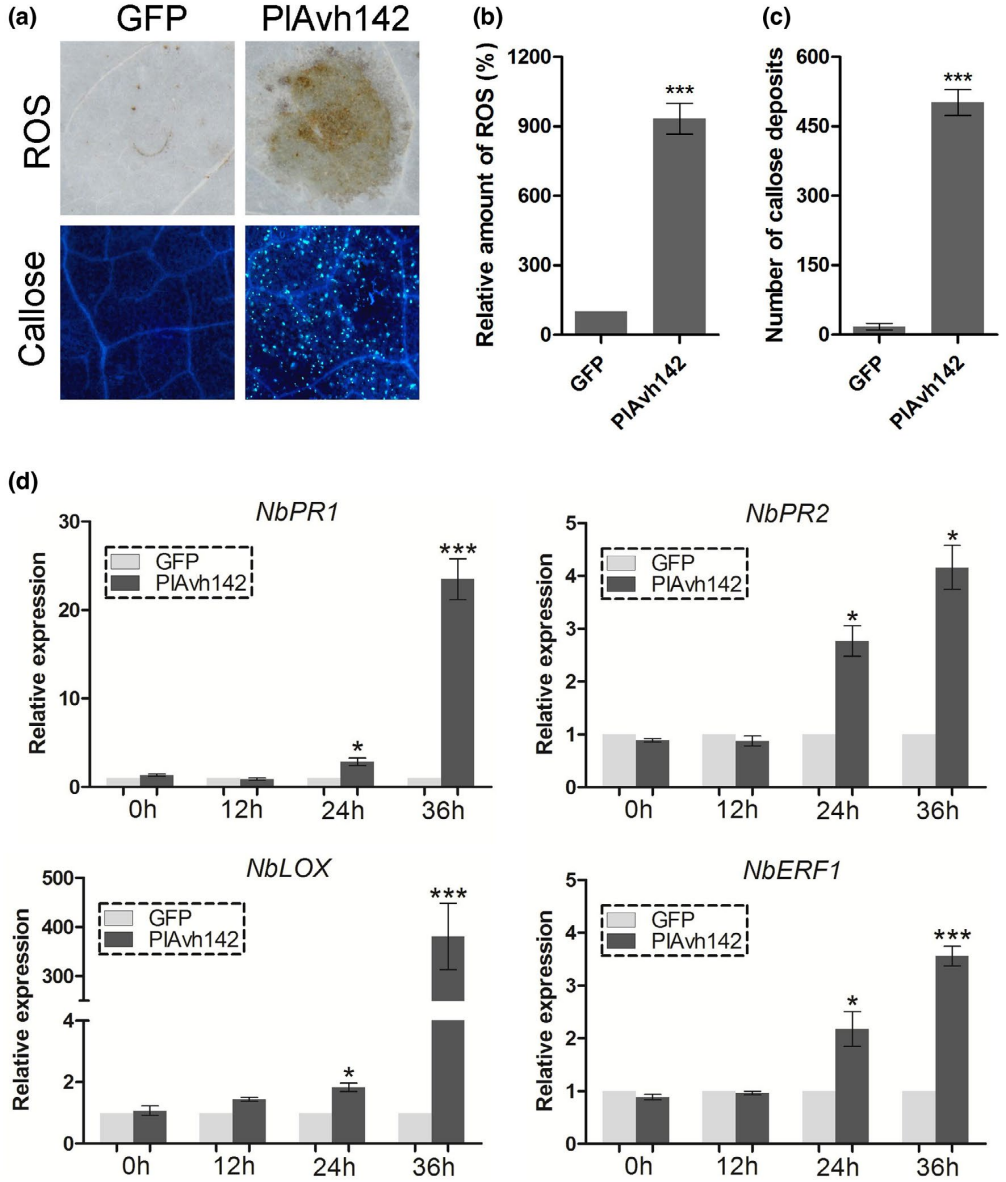
Transient expression of PlAvh142‐activated defence responses in *Nicotiana benthamiana*. (a) Reactive oxygen species (ROS) accumulation and callose deposition were observed in *PlAvh142‐*expressing plants at 36 hr post‐agroinfiltration (hpa). Representative images present the results obtained from three independent experiments. (b) Quantification of the ROS using ImageJ software. Mean ± *SE* was derived from three independent biological repeats (*n* ≥ 10), and *** denotes significant differences from the green fluorescent protein (GFP) (Student's *t* test: *p* < .01). (c) Quantification of the callose deposits per microscopic field using ImageJ software. Mean ± *SE* was derived from three independent biological repeats (*n* ≥ 10), and *** denotes significant differences from the GFP (Student's *t* test: *p* < .01) (D) Relative transcript levels of defence‐related genes in *N. benthamiana*. Leaves were harvested for RNA extraction of PlAvh142 or GFP control at 0, 12, 24, and 36 hpa. Transcript levels of candidate genes in PlAvh142‐treated leaves were normalized to that of GFP control, which was arbitrarily set as 1. The constitutively expressed gene *NbEF1α* was used as internal reference, according to the 2^−ΔΔ^ *^C^* ^t^ method. Data represent means ± *SD* from three independent biological repeats, asterisks denote significant differences from the control group (Student's *t* test: *p < .05; ***p < .01)

Cell death triggered by ETI is often considered as a part of the defence response resulting in suppression of disease progress (Balint‐Kurti, 2019). In general, some other immune responses may precede this exhibition, including ROS accumulation, callose deposition, and changes in levels of phytohormones (Asai and Yoshioka, 2009; Deb *et al.*, 2018; Bürger and Chory, 2019). Therefore, we assessed whether PlAvh142 could trigger oxidative burst production or callose deposition in *N. benthamiana* leaves via agroinfiltration, with expression of GFP alone as control. There was strong ROS accumulation and massive callose deposition in the PlAvh142‐expressing leaves at 36 hr post‐agroinfiltration (hpa) (Figure 2a–c). In contrast, the control leaves expressing GFP showed no visible ROS accumulation or callose deposition (Figure 2a–c). To further explore whether PlAvh142 is able to alter hormone signalling pathways in planta, the salicylic acid (SA)‐dependent defence pathway marker genes *NbPR1* and *NbPR2*, jasmonic acid (JA)‐dependent defence pathways marker gene *NbLOX*, and ethylene (ET)‐dependent defence pathways marker gene *NbERF1* (Dean *et al.*, 2005; Pieterse *et al.*, 2012; Zhang *et al.*, 2017) were chosen for quantitative reverse transcription polymerase chain reaction (RT‐qPCR) analysis at different time points (0, 12, 24, and 36 hr) after agroinfiltration of *N. benthamiana* leaves with GFP‐PlAvh142 or GFP. We found that *NbPR1*, *NbPR2*, *NbLOX*, and *NbERF1* were significantly up‐regulated in the PlAvh142‐expressing leaves at 24 and 36 hpa, compared with the GFP‐expressing samples (Figure 2d), indicating an induction of phytohormone signalling by PlAvh142 in *N. benthamiana*. Overall, our results suggested that the expression of PlAvh142 can activate various defence responses in planta.

### 2.3 The internal repeats are indispensable for PlAvh142‐inducing cell death

**Figure 3:**
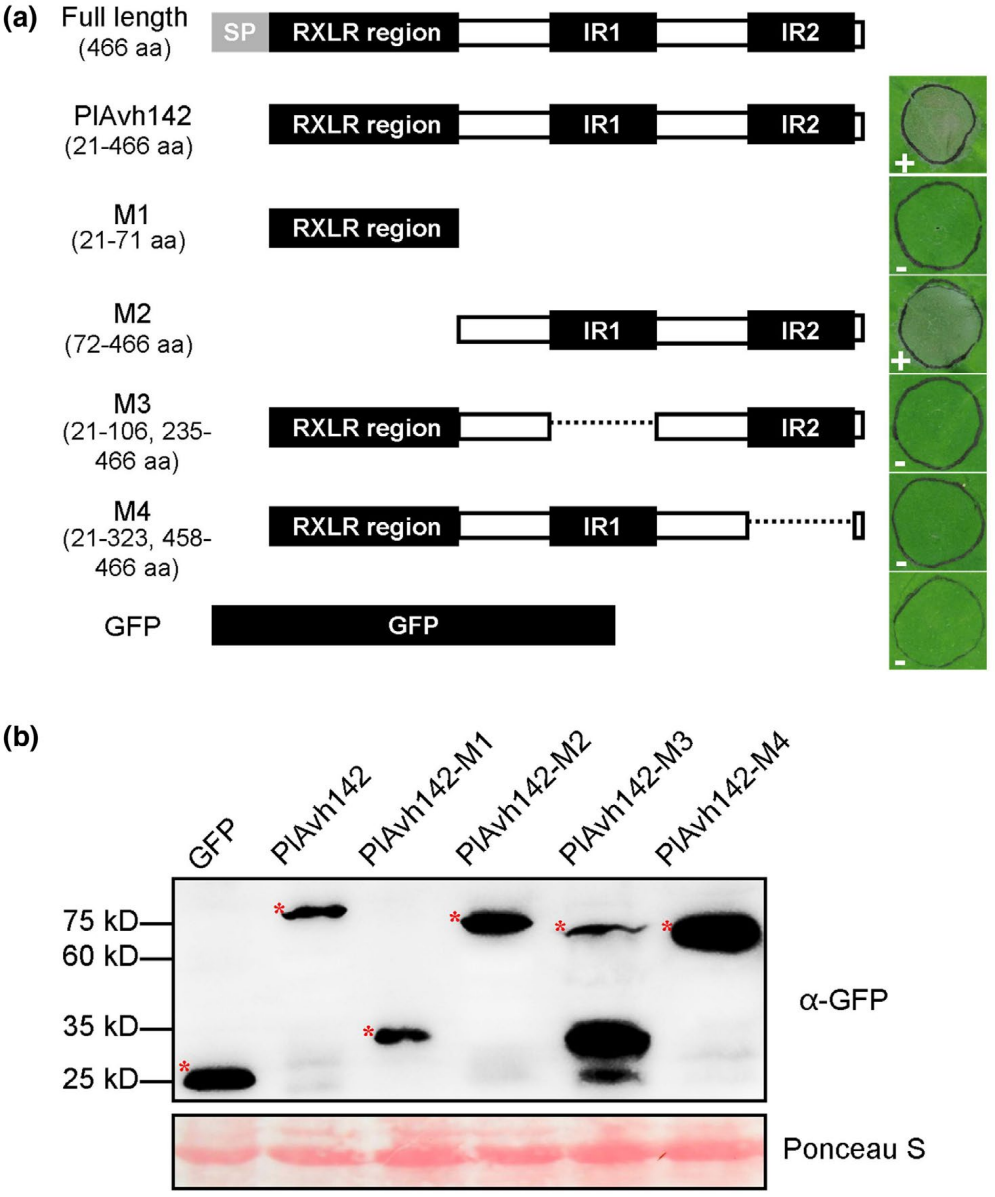
Deleting either of the internal repeats in PlAvh142 abolished the ability to trigger cell death. (a) Schematic diagrams of the protein structures of the PlAvh142 deletion mutants are shown in the left. Cell death symptoms in *Nicotiana benthamiana* leaves expressing PlAvh142 deletion mutants are shown in the right. Photographs were taken at 5 days post‐agroinfiltration. (b) Western blot confirmation of expression of PlAvh142 mutants using anti‐GFP (green fluorescent protein) antibody. The red asterisks indicate protein bands of the correct size. Protein loading was indicated by Ponceau S staining. Similar results were obtained from three independent experiments

W, Y or L motifs exist in some RXLR effectors (Win *et al.*, 2012); however, none of them was detected in PlAvh142 (Ye *et al.*, 2016). To further dissect the functions of PlAvh142, the conserved protein domain was analysed and predicted by the web‐based program Simple Modular Architecture Research Tool (SMART, http://smart.embl-heidelberg.de/). The prediction results show that in addition to the RXLR region, PlAvh142 comprises two internal repeats (IRs), IR1 (107–234 amino acids) and IR2 (324–457 amino acids), in its C‐terminus (Figures 3a and S1). The alignment of IR1 and IR2 showed 25% identity and 52% similarity by BLAST analysis performed on NCBI (https://blast.ncbi.nlm.nih.gov/Blast.cgi). To analyse the role of RXLR and IR motifs in cell death‐inducing activity, four truncated PlAvh142 variants were constructed and transiently expressed in *N. benthamiana* (Figure 3a,b). Our results show that M2 that lacked the RXLR region was still capable of inducing cell death, while either the IR1 (M3) or IR2 (M4) deletion resulted in loss of the ability to induce cell death in *N. benthamiana* (Figure 3a). These experiments suggest that the IRs are indispensable for its cell death induction, but not the RXLR region.

### 2.4 Cytoplasmic localization is critical for PlAvh142‐triggered cell death

**Figure 4:**
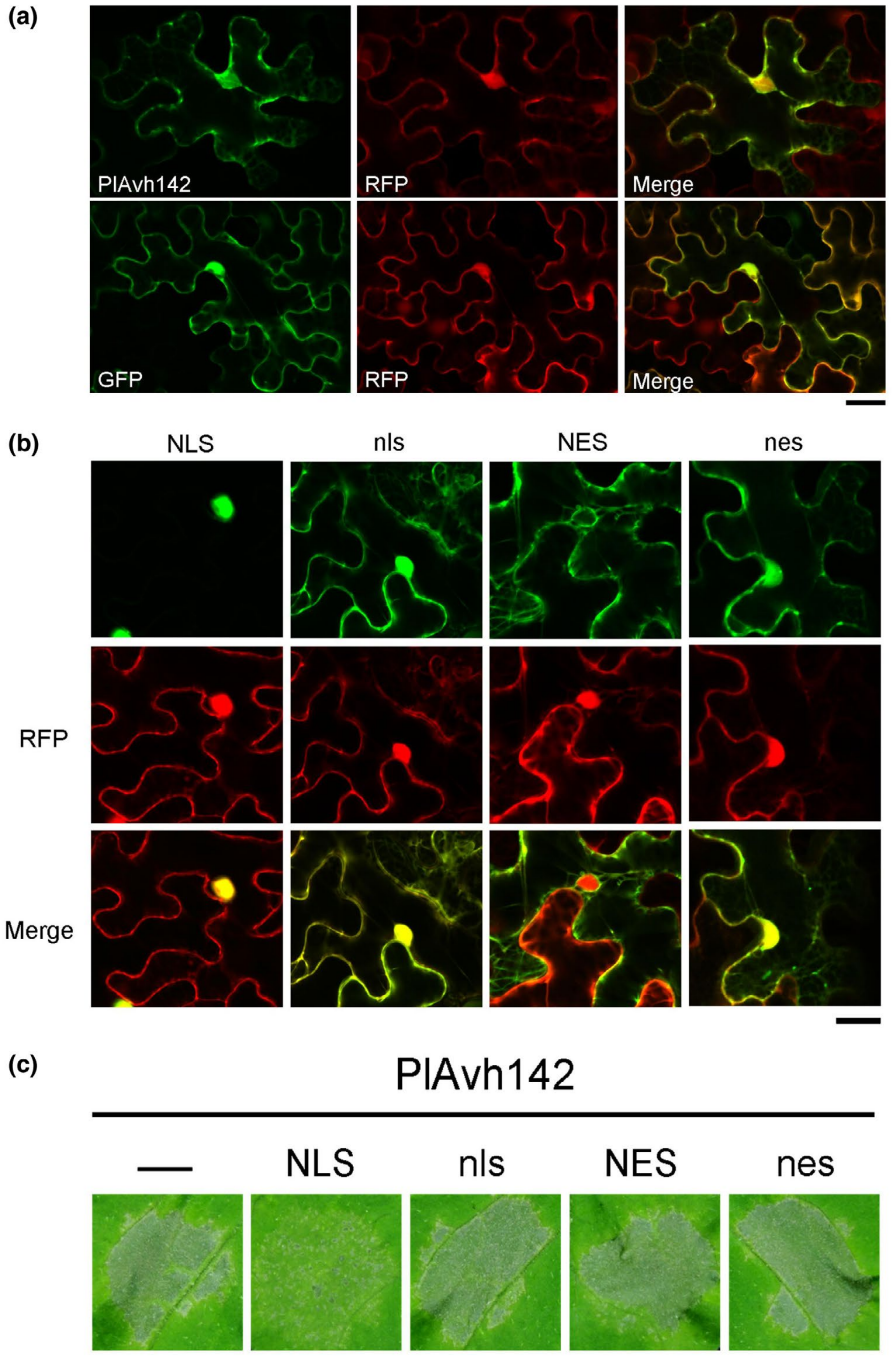
Cytoplasmic localization is critical for PlAvh142‐induced cell death. (a) Confocal microscopy imaging shows that green fluorescent protein (GFP)‐tagged PlAvh142 localizes to both the nucleus and the cytoplasm. GFP‐tagged PlAvh142 was transiently coexpressed with red fluorescent protein (RFP) via agroinfiltration in *Nicotiana benthamiana* with an OD_600_ of 0.1. Images are from GFP channel (left panel), RFP channel (middle panel), and the overlay (right panel) in *N. benthamiana* leaf cells. Scale bar, 20 μm. Photographs were taken at 36 hr post‐agroinfiltration (hpa). (b) Confocal microscopy images showing the subcellular localization of PlAvh142 attached with the nuclear localization signal (NLS) and nuclear export signal (NES), and the mutant forms nes and nls. The fusion constructs were agroinfiltrated at a final OD_600_ of 0.1. Photographs were taken at 36 hpa. Scale bars, 20 μm. (c) PlAvh142 could still induce cell death when localized to the cytoplasm. Strains with NLS‐, NES‐, nls‐ or nes‐tagged PlAvh142 were agroinfiltrated at a final OD_600_ of 0.1. Cell death triggered by NLS‐targeted PlAvh142 is delayed and weak. Photographs were taken at 5 days post‐agroinfiltration. Representative images for each construct were selected from three biological repeats, each of which contained at least five leaves for agroinfiltration

To gain insight into the subcellular localization of PlAvh142 in the plant cell, N‐terminal GFP‐tagged PlAvh142 (without the signal peptide) was transiently expressed in *N. benthamiana* leaves, and fluorescence was observed by confocal microscopy. The free monomeric red fluorescent protein (RFP) was coexpressed with GFP‐PlAvh142 and used as a marker to delineate the nucleus and cytoplasm. GFP‐PlAvh142 fusion protein could be detected in both cytoplasm and nucleus of plant cells (Figure 4a). It is documented that the cell death‐inducing activity of effectors is often determined by its subcellular location (Du *et al.*, 2015). Hence, to evaluate which subcellular localization of PlAvh142 is essential for the cell death induction, we forced GFP‐PlAvh142 to the nucleus or cytoplasm by attaching a nuclear localization signal (NLS) or nuclear export signal (NES), and assessed their cell death‐inducing activity, respectively. Constructs with mutated NLS (nls) or NES (nes) fused to GFP‐PlAvh142 were included as controls. The green fluorescence signals from NES‐fused or NLS‐fused GFP‐PlAvh142 were detected only in cytoplasm or nucleus, respectively, whereas mutated nls or nes fused GFP‐PlAvh142 showed similar fluorescence patterns to GFP‐PlAvh142 (Figure 4b). When these PlAvh142 variants were overexpressed in *N. benthamiana* leaves, GFP‐NES‐PlAvh142 showed strong cell death as the GFP‐PlAvh142 (Figure 4c). In contrast, the cell death was largely attenuated in the leaves expressing GFP‐NLS‐PlAvh142 (Figure 4c). Moreover, mutated nes and nls did not alter PlAvh142‐induced cell death (Figure 4c). Together, these results imply that localization of PlAvh142 in plant cell cytoplasm is critical for its cell death‐inducing activity.

### 2.5 *RAR1*, *SGT1*, and *HSP90* are required for PlAvh142‐induced cell death in *N. benthamiana*

**Figure 5:**
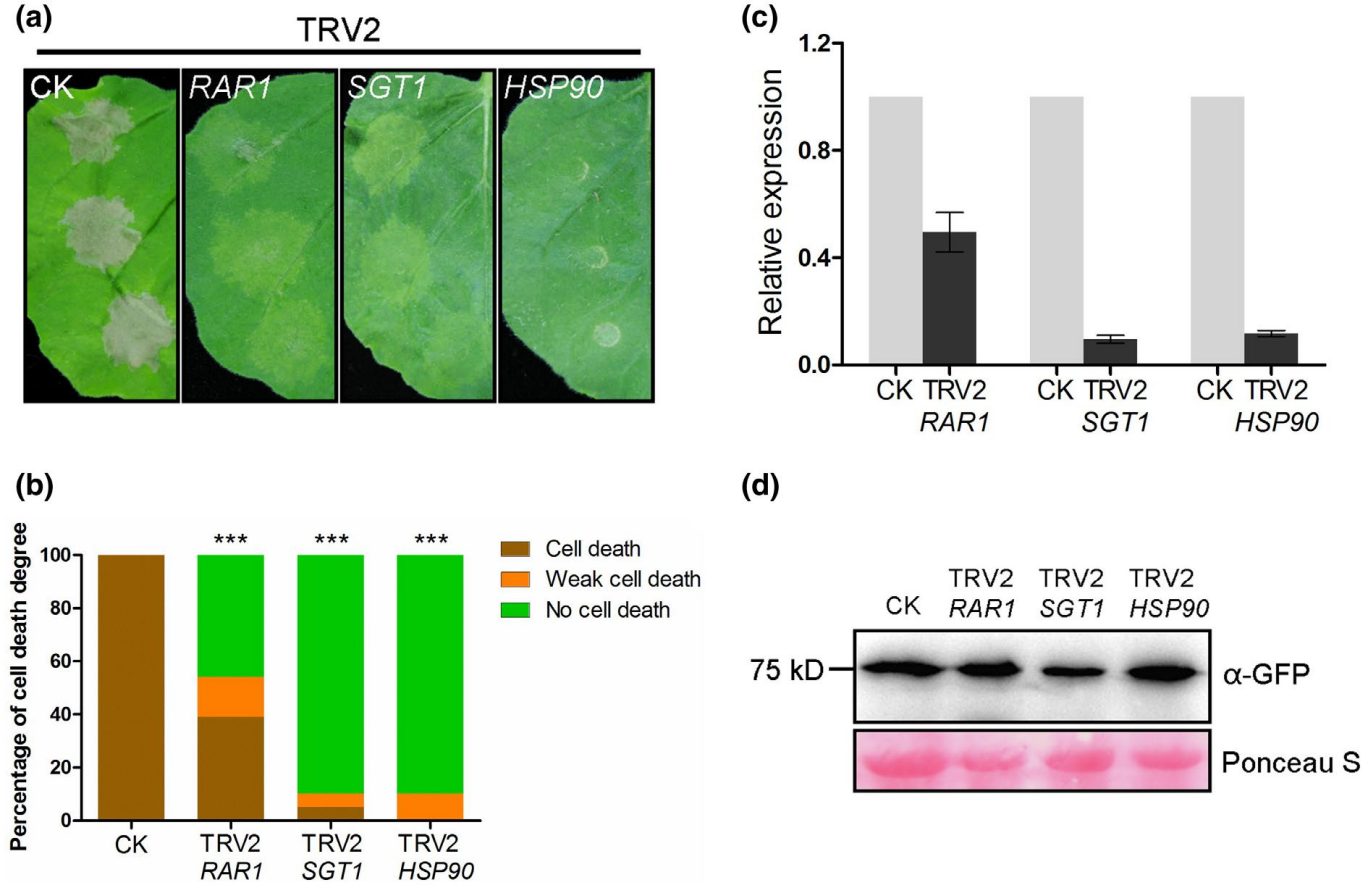
*RAR1*, *SGT1*, and *HSP90* were required for cell death induced by PlAvh142. (a) Representative photographs of PlAvh142‐induced cell death in silenced *Nicotiana benthamiana* leaves at 5 days post‐agroinfiltration (dpa). *Agrobacterium tumefaciens* carrying PlAvh142 was infiltrated into the upper leaves of silenced plants at 16–20 dpa of tobacco rattle virus (TRV) constructs. CK, unsilenced control. (b) Quantification of cell death in *N. benthamiana* leaves scored at 5 dpa. The degree of cell death was divided into three levels: no cell death, weak cell death, and strong cell death. Asterisks indicate significant differences from green fluorescent protein (GFP)‐silenced plants (Wilcoxon rank‐sum test: ***p < .001). (c) The transcript abundance of *RAR1*, *SGT1*, and *HSP90* in corresponding silenced plants was analysed by quantitative reverse transcription PCR (RT‐qPCR). The constitutively expressed gene *NbEF1α* was used as internal reference. Error bars represent the *SD* of three biological replicates. (d) Western blot of PlAvh142 in silenced leaves using the anti‐GFP antibody. Protein loading is indicated by Ponceau S staining. Similar results were obtained from three independent experiments

ETI mediated by intracellular immune receptors usually involves a set of downstream components (Chiang and Coaker, 2015). For example, *HSP90*, *SGT1*, *RAR1*, *NDR1*, and *EDS1* are reported to be associated with signal transduction in this process (Shirasu, 2009; Bhattacharjee *et al.*, 2011; Knepper *et al.*, 2011). In order to determine whether these plant innate immunity components are associated with PlAvh142‐induced cell death, virus‐induced gene silencing was used to individually knock down these genes in *N. benthamiana*. Two weeks after inoculation with *Agrobacterium* carrying the VIGS constructs, PlAvh142 was expressed in these silenced plants and then cell death was scored 5 days later. We observed that silencing of the *RAR1*, *SGT1*, or *HSP90* significantly compromised the cell death induced by PlAvh142 (Figure 5a,b). However, the cell death proportion in *NDR1*‐ or *EDS1*‐silenced plants was similar to that of the control plants (Figure S2a,b). The relative expression of these genes was verified by RT‐qPCR (Figures 5c and S2c). Immunoblotting assays confirmed the stable expression of GFP‐PlAvh142 in the *RAR1*, *SGT1*, or *HSP90*‐silenced plants (Figure 5d).

Other signalling pathway components of plant innate immunity, including *BAK1*, *SOBIR1*, *MEK1*, *MEK2*, *MAP3Kα*, *WIPK*, *SIPK*, *WRKY2*, and *MYB1*, were also individually knocked down in *N. benthamiana*. Nevertheless, there was no obvious difference in cell death proportion in these silenced plants compared with the control (Figure S2). Taken together, these results show that *RAR1*, *SGT1*, and *HSP90* are required for the cell death induced by PlAvh142 in *N. benthamiana*.

### 2.6 *PlAvh142* is up‐regulated in *P. litchii* zoospores and during the early phase of infection

**Figure 6:**
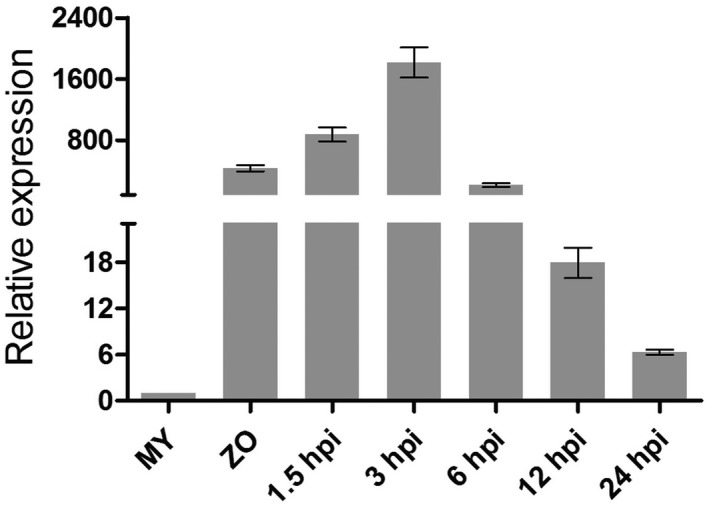
Expression profile of *Peronophythora litchii* RXLR effector gene *PlAvh142*. The relative transcript levels of *PlAvh142* during different development and infection stages of *P. litchii* were assessed by quantitative reverse transcription PCR (RT‐qPCR). MY, *P. litchii* mycelia grown in CJA medium; ZO, zoospores. Litchi leaves inoculated with *P. litchii* zoospores were harvested at 1.5, 3, 6, 12, and 24 hr post‐inoculation (hpi). The relative expression level was calibrated to the levels for the MY set as 1. The constitutively expressed gene *PlActin* was used as internal reference. Error bars represent the *SD* of three biological replicates

In order to investigate the biological function of *PlAvh142* in *P. litchii* development and pathogenicity, we assessed the expression profile of *PlAvh142* during different stages including mycelial growth, zoospore development, and infection of litchi leaf. The results showed that *PlAvh142* was highly up‐regulated in zoospores and infection stage (at 1.5, 3, 6, 12 or 24 hr post‐inoculation [hpi]) in comparison to mycelia. The highest expression peak appeared at 3 hpi, and then rapidly declined (Figure 6). However, the relative expression level at 24 hpi was still several fold higher than that of the mycelia. The accumulation of *PlAvh142* transcript in zoospores and infection phase suggests that it might play a role in *P. litchii* infection.

### 2.7 *PlAvh142* contributes to *P. litchii* virulence

**Figure 7:**
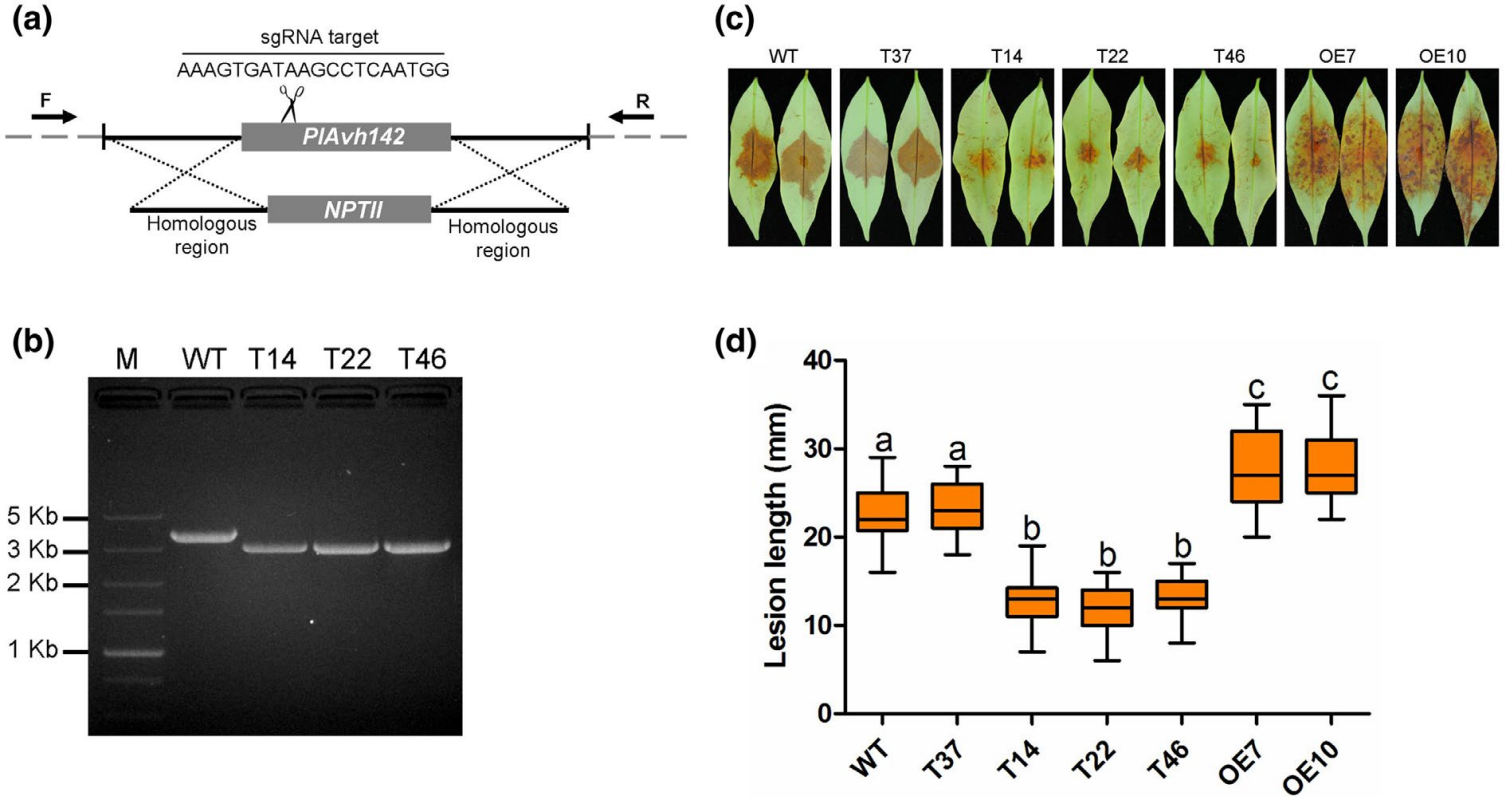
Knockout of *PlAvh142* in *Peronophythora litchii* attenuated the virulence to litchi. (a) Schematic diagram of the gene replacement at the *PlAvh142* locus by CRISPR/Cas9. The primers (F and R) used for PCR analysis are indicated by the horizontal arrows. (b) PCR analysis of the *PlAvh142* knockout mutants. Wild‐type (WT): 1,401 bp (*PlAvh142*) + 2,134 bp; knockout mutants (T14, T22, and T46): 795 bp (*NPTII*) + 2,134 bp. (c) Pathogenicity assays of WT and *PlAvh142* mutants on litchi leaves. Tender leaves were inoculated with 100 zoospores from WT, T37 (negative control), three *PlAvh142* knockout mutants T14, T22, and T46, and two overexpression mutants OE7 and OE10. Disease symptoms were visually monitored over a period of 48 hr and photographs were taken at 48 hr post‐inoculation (hpi). (d) Lesion length was measured after 48 hpi. Different letters represent significant differences (*p* < .01; Duncan's multiple range test). Bars represent medians and boxes the 25th and 75th percentiles. There were 30 leaves used in each of the three biological replicates

To further explore the possible role played by *PlAvh142* during *P. litchii* infection to its native host litchi, we generated the *PlAvh142* knockout mutants using the CRISPR/Cas9 gene editing system. The single guide (sg) RNA targeted *PlAvh142* were designed using the web tool EuPaGDT (http://grna.ctegd.uga.edu/) and gene replacement strategy schematically displayed in Figure 7a. Finally, we successfully generated three mutants, T14, T22, and T46, in which *PlAvh142* was replaced by the *NPTII* gene as verified by PCR amplification (Figure 7b) and confirmed by Sanger sequencing. At the same time, two *PlAvh142*‐overexpression mutants (OE7 and OE10) were also obtained and verified by RT‐qPCR (Figure S3). The mycelial growth of all mutants mentioned above was identical to that of wild‐type strain SHS3 (Figure S4).

Next, we inoculated the tender leaves of litchi plants with 100 zoospores from the wild‐type strain, knockout mutants, or overexpressed mutants. An unsuccessful knockout transformant T37 was included, serving as negative control. We observed that the *PlAvh142* knockout mutants caused fewer disease symptoms in litchi leaves at 48 hpi compared to that of the wild‐type strain (Figure 7c,d). In contrast, the overexpressed mutants caused more severe disease symptoms (Figure 7c,d). These results indicate that *PlAvh142* contribute to the *P. litchii* virulence during infection in its native host litchi.

## 3 DISCUSSION

Like other biotrophic and hemibiotrophic oomycete or fungal pathogens, *P. litchii* must overcome plant defences to establish host colonization. Pathogen effectors play important roles in subverting plant immunity. In oomycetes, much attention has been focused on identification and functional analysis of RXLR effectors over the past decade (Wang *et al*., 2011, 2019; Xiong *et al.*, 2014; Huang *et al.*, 2019). Although the genome sequence of *P. litchii* has been published with 245 RXLR predicted (Ye *et al.*, 2016), the function of these effectors during *P. litchii* infection remains unknown. In this study, we used the *N. benthamiana* model system for systematic screening of *P. litchii* RXLR effectors with plant cell death‐inducing activity. Here we reported that three effectors identified were able to trigger cell death in *N. benthamiana*, among which PlAvh142 could trigger plant cell death in a broad spectrum of plants and therefore it was chosen for further investigation. Unfortunately, we failed to test whether PlAvh142 could trigger cell death in its host plant, as we were not successful in a particle bombardment assay on litchi leaves despite repeated attempts.

Bioinformatic analysis showed that about 15% of the 245 predicted *P. litchii* RXLR effectors harbour IRs (Table S1), and PlAvh142 contains two IRs in its C‐terminal region. IRs exist widely in both eukaryotes and prokaryotes, and are considered to be involved in protein–protein interaction (Andrade *et al.*, 2001; Pawson and Nash, 2003; Björklund *et al.*, 2006). A recent report showed that *P. sojae* RXLR effector PsAvh23 contains two IRs and at least one IR that is required for its interaction with host target protein (Kong *et al.*, 2017). In this study we proved that both IRs in PlAvh142 were required for its cell death‐inducing activity. To our knowledge, this is the first report on the requirement of IRs for RXLR inducing plant cell death. However, it remains unclear whether these two IRs are required for interaction (if any) between PlAvh142 and its target protein(s) in the plant cells.

RXLR effectors could localize in different compartments of the host cells, in correspondence to their various molecular/cellular functions during the host–pathogen interaction (Liu *et al.*, 2018; Wang *et al.*, 2019a). Thus, perception of effector proteins by the cognate receptor(s) is frequently associated with their localized position, for example activation of R1‐mediated HR and resistance required localization of the R1/AVR1 pair in the nucleus, although both AVR1 and its cognate R protein R1 could be observed in cytoplasm and nucleus (Du *et al*., 2015). In this study we found that localization of PlAvh142 in the cytoplasm of the plant cell was sufficient and essential for inducing cell death, which is different from many other identified cell death‐inducing RXLR effectors shown to be localized to the plasma membrane or nucleus in plant cells (Yu *et al.*, 2012; Asai *et al.*, 2018; Yin *et al.*, 2019). A weak cell death was observed in plant cells expressing the nuclear localized PlAvh142 variant, which may be due to a small amount of protein residue in the cytoplasm. We infer that the cell death triggered by PlAvh142 effector may depend on its recognition in the cytoplasm of the plant cell.

A conserved chaperone complex consisting of HSP90, SGTI, and RAR1 is known to stabilize and sustain NLR‐mediated ETI responses (Azevedo *et al.*, 2002; Shirasu, 2009), and is required for plant cell death triggered by PvAvh74 (Yin *et al.*, 2019). *SGT1* and *HSP90*, rather than *RAR1,* are required for PpE4‐triggered cell death (Huang *et al.*, 2019). In the cases of *P. infestans* AVR‐blb2 and PITG_22798, cell death‐inducing activity is dependent on *SGT1* (Oha *et al.*, 2014; Wang *et al.*, 2017). Besides, *SGT1* is also involved in PTI and plant defence against viruses (Huitema *et al.*, 2005; Boter *et al.*, 2007). In the present study, silencing of *RAR1*, *SGT1*, and *HSP90* in *N. benthamiana* resulted in abolishing PlAvh142‐inducing cell death, suggesting that PlAvh142‐triggered cell death is possibly the consequence of plant perception and mediated by the HSR (HSP90, SGTI, and RAR1) complex. However, we silenced the other two well‐known components, *EDS1* and *NDR1*, of NLR signal transduction in *N. benthamiana*, and found no change in plant cell death induced by PlAvh142. Likewise, neither did we find any of the tested MAP kinases or transcription factors involved in the PlAvh142‐induced cell death. These results corroborate the fact that plants have multiple pathways for mediating cell death in response to different effectors. Combining the results of VIGS assays and PlAvh142‐induced cell death in various plants, we infer that a conserved recognition mechanism or function may underlie this effector. We speculate that either PlAvh142 targets a conserved and critical plant protein guarded by NLR genes or direct NLR recognition is conserved in various plants. Despite this, we cannot rule out the possibility that cell death triggered by PlAvh142 is mediated by an unknown mechanism.

Hemibiotrophic pathogens need to keep the host cell alive before establishing their colonization in the biotrophic stage, and later trigger cell death to promote the necrotrophic infection (Qutob *et al.*, 2002). Some apoplastic or cytoplasmic effectors from *Phytophthora* pathogens display elicitor activity, that is, they could trigger plant immunity responses, and concurrently contribute to the virulence or promote pathogen colonization. Examples include PsXEG1 (Ma *et al.*, 2015, 2017; Wang *et al.*, 2018b), Avh238 (Yang *et al.*, 2017), and PpE4 (Huang *et al.*, 2019). Another similar example was reported in the cell death‐inducing RXLR effector, PcAvh1, which is a virulence factor of *P. capsici* as its deletion mutants displayed reduced pathogenicity in contrast to the more aggressive overexpression mutants (Chen *et al.*, 2019). Such a seemingly contradictory phenomenon was also observed for PlAvh142 in this study, and we raise the following hypotheses in an attempt to explain this. First, the accumulation of PlAvh142 protein during *P. litchii* infection to the native host plant may be insufficient to trigger cell death and/or immune responses under natural conditions. Second, cell death triggered by one RXLR effector could be suppressed by the other cooperative effectors during pathogen infection, which has been already reported (Wang *et al.*, 2011). Therefore, PlAvh142 is still able to enhance colonization and execute its virulence function when its elicitor activity is blocked. Alternatively, the cell death induced by PlAvh142 may contribute to the transition from biotrophy to necrotrophy and thus positively regulate *P. litchii* virulence. Although it is generally accepted that RXLR effectors facilitate pathogen infection mainly by modulating plant immune system, the functional relationship (if any) between PlAvh142’s cell death‐inducing activity and contribution to virulence awaits elucidation; alternatively, a mechanism(s) other than cell death induction underlying PlAvh142 virulence function needs to be uncovered.

Overall, we report here, for the first time, that an RXLR effector secreted by *P. litchii* acts as an elicitor that triggers immune responses in plants*.* The possible mechanism involved in perception of PlAvh142 will be very useful for exploring the potential resistance genes or materials for the litchi plant, which provides insights into novel disease control strategies. The next step is to identify the potential PlAvh142‐interacting protein(s) to reveal the functions of RXLR effector in litchi–*P. litchii* interaction, for a better understanding of the biological functions of RXLR effectors.

## 4 EXPERIMENTAL PROCEDURES

### 4.1 Microbial strains, plant material, and culture conditions

*P. litchii* strain SHS3 was cultured on carrot juice agar (CJA) medium (juice from 200 g carrot topped up to 1 L, 15 g agar/L for solid medium) at 25 °C in the dark. *Escherichia coli* DH5α was cultured at 37 °C in Luria Bertani (LB) medium and used for cloning and propagation of recombinant plasmids. *A. tumefaciens* GV3101 used for transient expression was cultured at 28 °C in LB medium using appropriate antibiotics. *N. benthamiana* was maintained in the greenhouse at 22–25 °C with a photoperiod of 18 hr light/6 hr darkness.

### 4.2 Plasmid construction

All the primers used in this study are listed in Table S2. The PCR fragments were amplified by Phanta Max Super‐Fidelity DNA Polymerase (Vazyme). For pVX, vectors and gene (without signal peptide) fragments were digested by *Sma*I and *Not*I (New England Biolabs) in the appropriate conditions. The digested fragments were linked to the linearized pVX by T4 DNA ligase (Takara). For pBINGFP2 and pTRV2, vectors and gene fragments (without signal peptide for pBINGFP2) were digested by *Sma*I and *Eco*RI, respectively (New England Biolabs) in the appropriate conditions. The fragments were linked to the linearized pBINGFP2 by ClonExpress MultiS One Step Cloning Kit (Vazyme). To construct the overexpression vector, the full length of PlAvh142 coding sequence was linked to the linearized pTORmRFP4, which were digested by *Cla*I and *Bsi*WI (New England Biolabs). The vectors pYF2.3G‐RibosgRNA and pBluescript II KS used for knockout of PlAvh142 by CRISPR/Cas9 were generated as described previously (Fang and Tyler, 2016).

### 4.3 RNA extraction, cDNA synthesis, and expression analysis of *PlAvh142*

Mycelia and litchi leaves infected with zoospores suspension of *P. litchii* were harvested at the indicated time points and RNA was extracted using All‐In‐One DNA/RNA Mini‐preps Kit (Bio Basic) according to the recommended protocol. All cDNAs were synthesized from total RNA by PrimeScript RT Master Mix (Takara). RT‐qPCR was performed in 20 μl reactions that included 20 ng cDNA, 0.4 μΜ gene‐specific primer of *PlAvh142*, 10 μl SYBR Premix Ex Taq II (Takara) and 6.4 μl dH_2_O. The RT‐qPCRs were performed on qTOWER^3^ Real‐Time PCR thermal cyclers (Analytik Jena) under the following conditions: 95 °C for 2 min, 40 cycles at 95 °C for 30 s, and 60 °C for 30 s to calculate cycle threshold values, followed by a dissociation programme of 95 °C for 15 s, 60 °C for 1 min, and 95 °C for 15 s to obtain melt curves. The relative expression values were determined using *Actin* from *P. litchii* as reference gene and calculated with the formula 2^−ΔΔ^ *^C^* ^t^.

### 4.4 Agroinfection assay in *N. benthamiana*

The *PlAvh142* gene was amplified from *P. litchii* cDNA and then cloned into the PVX vector pGR107 and pBINGFP2, respectively. The recombinant plasmid was introduced into *A. tumefaciens* GV3101 by heat shock. For cell death induction experiments, *A. tumefaciens* carrying the respective recombinant plasmids was cultured in LB medium at 28 °C with shaking at 200 rpm for 48 hr. The cultures were harvested and washed three times with 10 mM MgCl_2_, then resuspended in 10 mM MgCl_2_ to achieve final concentrations before agroinfiltration in *N. benthamiana* leaves. Symptoms were visually monitored and photographs were taken after 3–8 days. The experiments were repeated at least three times.

### 4.5 Callose and ROS staining

To observe callose deposition and ROS accumulation in planta, the whole leaves of *N. benthamiana* were harvested at 36 hpa. For callose deposition assay, leaves were stained with 0.01% aniline blue in 150 mM K_2_HPO_4_ buffer 1–2 hr after destaining in 96% ethanol (Sohn *et al.*, 2007) and subsequently imaged by Olympus BX53 microscopy system. For ROS accumulation assays, leaves were visualized using diaminobenzidine‐HCl solution (1 mg/ml, pH 3.8) in darkness for 8–12 hr and subsequently destained with 96% ethanol (Zhang *et al.*, 2017). The quantification of callose deposition and ROS accumulation was calculated using ImageJ software. At least three leaves were tested in each independent experiment. The experiments were repeated at least three times.

### 4.6 Virus‐induced gene silencing assays in *N. benthamiana*

The cultured *A. tumefaciens* strains carrying pTRV2::*BAK1*, pTRV2::*SOBIR1*, pTRV2::*MEK1*, pTRV2::*MEK2*, pTRV2::*MAP3Kα*, pTRV2::*SIPK*, pTRV2::*WIPK*, pTRV2::*WRKY2*, pTRV2::*RAR1*, pTRV2::*HSP90*, pTRV2::*SGT1*, pTRV2::*NDR1*, pTRV2::*EDS1*, pTRV2::*MYB*, pTRV2::*GUS*, and pTRV1 were resuspended in the agroinfiltration buffer described above. Each pTRV2 and pTRV1 was mixed in equal ratios with a final OD_600_ of 0.2 for each. pTRV2::*GUS* was used as a control. The three primary leaves of 4‐leaf‐stage *N. benthamiana* plants were infiltrated by the mixtures. The gene silencing efficiency was analysed by RT‐qPCR and PlAvh142 was agroinfiltrated at 16–20 days after infiltration with *A*. *tumefaciens* carrying the VIGS constructs. The degree of cell death was monitored at 5 days after PlAvh142 was agroinfiltrated. The experiments were repeated at least three times.

### 4.7 Protein extraction and western blot analysis

The leaves of *N. benthamiana* that were infiltrated with *A. tumefaciens* were ground into powder in liquid nitrogen and vigorously mixed with 0.5 ml of precooled radioimmunoprecipitation assay buffer (RIPA buffer) (250 mM Tris‐HCl [pH 7.6], 150 mM NaCl, 1% NP‐40, 1% sodium deoxycholate, 0.1% sodium dodecyl sulphate [SDS] [Thermo Fisher Scientific]). After 5 min of incubation on ice, the samples were centrifuged at 14,000 × g for 15 min to obtain the supernatant. After adding loading buffer and boiling for 5 min, total proteins were separated by SDS‐polyacrylamide gel electrophoresis (SDS‐PAGE) gels. Then the proteins were transferred to polyvinylidene fluoride (PVDF) membranes (Bio‐Rad) followed by blocking in 5% non‐fat milk dissolved in PBST (phosphate‐buffered saline + 0.1% Tween 20). Mouse anti‐GFP monoclonal antibody (Abbkine) was used at 1:5,000 dilution to detect the corresponding fusion proteins. The membranes were washed three times with PBST and incubated with a goat anti‐mouse antibody (1:10,000) (Mei5 Biotechnology). Proteins were visualized by Efficient Chemiluminescence kit (Genview), and photographs were taken under the imaging system (Tanon).

### 4.8 Confocal microscopy

For fluorescence observations, patches of *N. benthamiana* leaves were cut after 2 dpa and used for confocal imaging on a Nikon A1 laser scanning microscope with a 40× objective lens. RFP or GFP fluorescence was observed at an excitation wavelength of 561 or 488 nm, respectively.

### 4.9 Transformation of *P. litchii*

To generate knockout and overexpressing transformants, *P. litchii* protoplasts were transformed using the polyethylene glycol (PEG)–CaCl_2_‐mediated method as described previously (Fang and Tyler, 2016; Jiang *et al.*, 2017). For knockout experiment, plasmids pYF2‐PsNLS‐hSpCas9, pYF2.3G‐RibosgRNA::*PlAvh142*, and pBluescript II KS::*PlAvh142* were cotransformed into protoplasts of strain SHS3. For the overexpression experiment, plasmid pTORmRFP4::*PlAvh142* was transformed into protoplasts of strain SHS3. The transformed protoplasts were regenerated overnight, and the recovered mycelia were selected on CJA medium with 30 μg/ml G418. After 2–3 days, the primary transformants were transferred to new selective medium, named sequentially and maintained for subsequent analyses.

### 4.10 Pathogenicity assays

For pathogenicity assays, zoospores were inoculated on the tender leaves of litchi (Guiwei), which were collected from the litchi orchard in South China Agricultural University, Guangzhou, Guangdong province. One hundred zoospores of each strain were inoculated on the center of the tender leaf, and kept at 80% humidity in 12 hr light/12 hr darkness at 25 °C. Each strain was tested on no fewer than 10 leaves. The symptoms were observed and the lesion diameter was measured at 48 hpi. The experiments were repeated at least three times.

## Supporting information


**FIGURE S1** Protein sequences of PlAvh142. Different regions are marked with bold lines

**FIGURE S2** Analysis of the components involved in PlAvh142‐induced cell death. *Nicotiana benthamiana* leaves were agroinfiltrated with pTRV2 constructs targeting *BAK1*, *SOBIR1*, *EDS1*, *NDR1*, *MEK1*, *MEK2*, *MAP3Kα*, *SIPK*, *WIPK*, *WRKY2,* and *MYB*; pTRV2::*GFP *was used as a control. (a) Representative images of PlAvh142‐induced cell death in silenced *N. benthamiana* leaves at 5 days post‐agroinfiltration (dpa). *Agrobacterium tumefaciens* carrying PlAvh142 was infiltrated into the upper leaves of silenced plants at 16–20 dpa of TRV constructs. (B) Quantification of cell death in *N. benthamiana* leaves scored at 5 dpa. The degree of cell death was divided into three levels: no cell death, weak cell death, and strong cell death. Asterisks indicate significant differences from green fluorescence protein (GFP)‐silenced plants (Wilcoxon rank‐sum test: ***, *p* < .001). (C) The transcript abundance of the genes in corresponding silenced plants was detected by RT‐qPCR. The constitutive expression gene *NbEF1α *was used as internal reference. Error bars represent the *SD* of three biological replicates. Similar results were obtained from three independent experiments

**FIGURE S3** The relative expression level of *PlAvh142*‐overexpressing mutants. RT‐qPCR was used to determine the overexpressing level of the mutants. The relative expression level was calibrated to the levels for the wild type that set as 1. The constitutive expression gene *PlActin *was used as internal reference. Error bars represent the *SD* of three biological replicates

**FIGURE S4** The phenotype of *PlAvh142* mutants were identical to wild type. (a) Colony morphology of *PlAvh142* mutants grown on carrot juice agar medium after 5 days. (b) Growth rates of *PlAvh142* mutants. Letters represent significant differences (*p *< .05; Duncan’s multiple range test). Similar results were obtained from three independent experiments

**TABLE S1** Screening of cell death‐inducing RXLR effectors in *Peronophythora* *litchii*

**TABLE S2** Primers used in this study

## Data Availability

The data that support the findings of this study are available from the corresponding author upon reasonable request.

## References

[mpp12905-bib-0001] Andrade, M.A. , Perez‐Iratxeta, C. and Ponting, C.P. (2001) Protein repeats: structures, functions, and evolution. Journal of Structural Biology, 134, 117–131. 11551174 10.1006/jsbi.2001.4392

[mpp12905-bib-0002] Asai, S. and Yoshioka, H. (2009) Nitric oxide as a partner of reactive oxygen species participates in disease resistance to necrotrophic pathogen *Botrytis cinerea* in *Nicotiana benthamiana* . Molecular Plant‐Microbe Interactions, 22, 619–629. 19445587 10.1094/MPMI-22-6-0619

[mpp12905-bib-0003] Asai, S. , Furzer, O.J. , Cevik, V. , Kim, D.S. , Ishaque, N. , Goritschnig, S. *et al* (2018) A downy mildew effector evades recognition by polymorphism of expression and subcellular localization. Nature Communications, 9, 5159. 10.1038/s41467-018-07469-3 PMC6281644 30518923

[mpp12905-bib-0004] Azevedo, C. , Sadanandom, A. , Kitagawa, K. , Freialdenhoven, A. , Shirasu, K. and Schulze‐Lefert, P. (2002) The RAR1 interactor SGT1, an essential component of R gene‐triggered disease resistance. Science, 295, 2073–3016. 11847307 10.1126/science.1067554

[mpp12905-bib-0005] Balint‐Kurti, P. (2019) The plant hypersensitive response: concepts, control and consequences. Molecular Plant Pathology, 20, 1163–1178. 31305008 10.1111/mpp.12821 PMC6640183

[mpp12905-bib-0006] Baxter, L. , Tripathy, S. , Ishaque, N. , Boot, N. , Cabral, A. , Kemen, E. *et al* (2010) Signatures of adaptation to obligate biotrophy in the *Hyaloperonospora arabidopsidis* genome. Science, 330, 1549–1551. 21148394 10.1126/science.1195203 PMC3971456

[mpp12905-bib-0007] Beakes, G.W. , Glockling, S.L. and Sekimoto, S. (2012) The evolutionary phylogeny of the oomycete “fungi”. Protoplasma, 249, 3–19. 21424613 10.1007/s00709-011-0269-2

[mpp12905-bib-0008] Bhattacharjee, S. , Halane, M.K. , Kim, S.H. and Gassmann, W. (2011) Pathogen effectors target *Arabidopsis* EDS1 and alter its interactions with immune regulators. Science, 334, 1405–1408. 22158819 10.1126/science.1211592

[mpp12905-bib-0009] Björklund, A.K. , Ekman, D. and Elofsson, A. (2006) Expansion of protein domain repeats. PLoS Computational Biology, 8, 114. 10.1371/journal.pcbi.0020114 PMC1553488 16933986

[mpp12905-bib-0010] Boter, M. , Amigues, B. , Peart, J. , Breuer, C. , Kadota, Y. , Casais, C. *et al* (2007) Structural and functional analysis of SGT1 reveals that its interaction with HSP90 is required for the accumulation of Rx, an R protein involved in plant immunity. The Plant Cell, 19, 3791–3804. 18032631 10.1105/tpc.107.050427 PMC2174866

[mpp12905-bib-0011] Boutrot, F. and Zipfel, C. (2017) Function, discovery, and exploitation of plant pattern recognition receptors for broad‐spectrum disease resistance. Annual Review of Phytopathology, 55, 257–286. 10.1146/annurev-phyto-080614-120106 28617654

[mpp12905-bib-0012] Bürger, M. and Chory, J. (2019) Stressed out about hormones: how plants orchestrate immunity. Cell Host & Microbe, 26, 163–172. 31415749 10.1016/j.chom.2019.07.006 PMC7228804

[mpp12905-bib-0013] Cesari, S. (2018) Multiple strategies for pathogen perception by plant immune receptors. New Phytologist, 219, 17–24. 29131341 10.1111/nph.14877

[mpp12905-bib-0014] Chen, X. , Zhang, Y. , Li, H. , Zhang, Z. , Sheng, G. , Li, Y. *et al* (2019) The RXLR effector PcAvh1 is required for full virulence of *Phytophthora capsici* . Molecular Plant‐Microbe Interactions, 32, 986–1000. 30811314 10.1094/MPMI-09-18-0251-R

[mpp12905-bib-0015] Chiang, Y. and Coaker, G. (2015) Effector triggered immunity: NLR immune perception and downstream defense responses. The Arabidopsis Book, 13, e183.

[mpp12905-bib-0016] Chisholm, S.T. , Coaker, G. , Day, B. and Staskawicz, B.J. (2006) Host–microbe interactions: shaping the evolution of the plant immune response. Cell, 124, 803–814. 16497589 10.1016/j.cell.2006.02.008

[mpp12905-bib-0017] Dean, J.D. , Goodwin, P.H. and Hsiang, T. (2005) Induction of *glutathione S‐transferase* genes of *Nicotiana benthamiana* following infection by *Colletotrichum destructivum* and *C. orbiculare* and involvement of one in resistance. Journal of Experimental Botany, 56, 1525–1533. 15837710 10.1093/jxb/eri145

[mpp12905-bib-0018] Deb, D. , Anderson, R.G. , Kin, T. , Tyler, B.M. and McDowell, J.M. (2018) Conserved RxLR effectors from oomycetes *Hyaloperonospora arabidopsidis* and *Phytophthora sojae* suppress PAMP‐ and effector‐triggered immunity in diverse plants. Molecular Plant‐Microbe Interactions, 31, 374–385. 29106332 10.1094/MPMI-07-17-0169-FI

[mpp12905-bib-0019] Dodds, P.N. and Rathjen, J.P. (2010) Plant immunity: towards an integrated view of plant–pathogen interactions. Nature Reviews Genetics, 11, 539–548. 10.1038/nrg2812 20585331

[mpp12905-bib-0020] Dou, D. , Kale, S.D. , Wang, X. , Jiang, R.H. , Bruce, N.A. , Arredondo, F.D. *et al* (2008) RxLR‐mediated entry of *Phytophthora sojae* effector Avr1b into soybean cells does not require pathogen‐encoded machinery. The Plant Cell, 20, 1930–1947. 18621946 10.1105/tpc.107.056093 PMC2518231

[mpp12905-bib-0021] Du, Y. , Berg, J. , Govers, F. and Bouwmeester, K. (2015) Immune activation mediated by the late blight resistance protein R1 requires nuclear localization of R1 and the effector AVR1. New Phytologist, 207, 735–747. 25760731 10.1111/nph.13355

[mpp12905-bib-0022] Fan, G. , Yang, Y. , Li, T. , Lu, W. , Du, Y. , Qiang, X. *et al* (2018) A *Phytophthora capsici* RXLR effector targets and inhibits a plant PPlase to suppress endoplasmic reticulum‐mediated immunity. Molecular Plant Pathology, 11, 1067–1083. 10.1016/j.molp.2018.05.009 29864524

[mpp12905-bib-0023] Fang, Y. and Tyler, B.M. (2016) Efficient disruption and replacement of an effector gene in the oomycete *Phytophthora sojae* using CRISPR/Cas9. Molecular Plant Pathology, 17, 127–139. 26507366 10.1111/mpp.12318 PMC6638440

[mpp12905-bib-0024] Franceschetti, M. , Maqbool, A. , Jimenez‐Dalmaroni, M.J. , Pennington, H.G. , Kamoun, S. and Banfield, M.J. (2017) Effectors of filamentous plant pathogens: commonalities amid diversity. Microbiology and Molecular Biology Reviews, 81, e00066–e116. 28356329 10.1128/MMBR.00066-16 PMC5485802

[mpp12905-bib-0025] Fry, W.E. , Birch, P.R. , Judelson, H.S. , Gruenwald, N.J. , Danies, G. , Everts, K.L. *et al* (2015) Five reasons to consider *Phytophthora infestans* a reemerging pathogen. Phytopathology, 105, 966–981. 25760519 10.1094/PHYTO-01-15-0005-FI

[mpp12905-bib-0026] Göker, M. , Voglmayr, H. , Riethmüller, A. and Oberwinkler, F. (2007) How do obligate parasites evolve? A multi‐gene phylogenetic analysis of downy mildews. Fungal Genetics and Biology, 44, 105–122. 16990040 10.1016/j.fgb.2006.07.005

[mpp12905-bib-0027] Haas, B.J. , Kamoun, S. , Zody, M.C. , Jiang, R.H.Y. , Handsaker, R.E. , Cano, L.M. *et al* (2009) Genome sequence and analysis of the Irish potato famine pathogen *Phytophthora infestans* . Nature, 461, 393–398. 19741609 10.1038/nature08358

[mpp12905-bib-0028] Han, G. (2018) Origin and evolution of the plant immune system. New Phytologist, 222, 70–83. 10.1111/nph.15596 30575972

[mpp12905-bib-0029] Huang, G. , Liu, Z. , Gu, B. , Zhao, H. , Jia, J. , Fan, G. *et al* (2019) An RXLR effector secreted by *Phytophthora parasitica* is a virulence factor and triggers cell death in various plants. Molecular Plant Pathology, 20, 356–371. 30320960 10.1111/mpp.12760 PMC6637884

[mpp12905-bib-0030] Huitema, E. , Vleeshouwers, V. , Cakir, C. , Kamoun, S. and Govers, F. (2005) Differences in intensity and specificity of hypersensitive response induction in *Nicotiana* spp. by INN, INF2A, and INF2B of *Phytophthora infestans* . Molecular Plant‐Microbe Interactions, 18, 183–193. 15782632 10.1094/MPMI-18-0183

[mpp12905-bib-0031] Ingle, R.A. , Carstens, M. and Denby, K.J. (2006) PAMP recognition and the plant–pathogen arms race. BioEssays, 28, 880–889. 16937346 10.1002/bies.20457

[mpp12905-bib-0032] Jiang, L. , Situ, J. , Deng, Y.Z. , Wan, L. , Xu, D. , Chen, Y. *et al* (2018) PIMAPK10, a mitogen‐activated protein kinase (MAPK) in *Peronophythora litchii*, is required for mycelial growth, sporulation, laccase activity, and plant infection. Frontiers in Microbiology, 9, 426. 29568294 10.3389/fmicb.2018.00426 PMC5852060

[mpp12905-bib-0033] Jiang, L. , Ye, W. , Situ, J. , Chen, Y. , Yang, X. , Kong, G. *et al* (2017) A Puf RNA‐binding protein encoding gene *PlM90* regulates the sexual and asexual life stages of the litchi downy blight pathogen *Peronophythora litchii* . Fungal Genetics and Biology, 98, 39–45. 27939344 10.1016/j.fgb.2016.12.002

[mpp12905-bib-0034] Jones, J.D. and Dangl, J.L. (2006) The plant immune system. Nature, 444, 323–329. 17108957 10.1038/nature05286

[mpp12905-bib-0035] Knepper, C. , Savory, E.A. and Day, B. (2011) The role of NDR1 in pathogen perception and plant defense signaling. Plant Signaling & Behavior, 6, 1114–1116. 21758001 10.4161/psb.6.8.15843 PMC3260705

[mpp12905-bib-0036] Kale, S.D. , Gu, B. , Capelluto, D.G. , Dou, D. , Feldman, E. , Rumore, A. *et al* (2010) External lipid PI3P mediates entry of eukaryotic pathogen effectors into plant and animal host cells. Cell, 142, 284–295. 20655469 10.1016/j.cell.2010.06.008

[mpp12905-bib-0037] Kong, G. , Wan, L. , Deng, Y.Z. , Yang, W. , Li, W. , Jiang, L. *et al* (2019) Pectin acetylesterase PAE5 is associated with the virulence of plant pathogenic oomycete *Peronophythora litchii* . Physiological and Molecular Plant Pathology, 106, 16–22.

[mpp12905-bib-0038] Kong, L. , Qiu, X. , Kang, J. , Wang, Y. , Chen, H. , Huang, J. *et al* (2017) A *Phytophthora* effector manipulates host histone acetylation and reprograms defense gene expression to promote infection. Current Biology, 27, 981–991. 28318979 10.1016/j.cub.2017.02.044

[mpp12905-bib-0039] Lamour, K.H. , Stam, R. , Jupe, J. and Huitema, E. (2012) The oomycete broad‐host‐range pathogen *Phytophthora capsici* . Molecular Plant Pathology, 13, 329–337. 22013895 10.1111/j.1364-3703.2011.00754.x PMC6638677

[mpp12905-bib-0040] Liu, Y. , Lan, X. , Song, X. , Yin, L. , Dry, L. , Qu, J. *et al* (2018) *In planta* functional analysis and subcellular localization of the oomycete pathogen *Plasmopara viticola* candidate RXLR effector repertoire. Frontiers in Microbiology, 9, 286. 29706971 10.3389/fpls.2018.00286 PMC5908963

[mpp12905-bib-0041] Ma, Z. , Song, T. , Zhu, L. , Ye, W. , Wang, Y. , Shao, Y. *et al* (2015) A *Phytophthora sojae* Glycoside Hydrolase 12 protein is a major virulence factor during soybean infection and is recognized as a PAMP. The Plant Cell, 27, 2057–2072. 26163574 10.1105/tpc.15.00390 PMC4531360

[mpp12905-bib-0042] Ma, Z. , Zhu, L. , Song, T. , Wang, Y. , Zhang, Q. , Xia, Y. *et al* (2017) A paralogous decoy protects *Phytophthora sojae* apoplastic effector PsXEG1 from a host inhibitor. Science, 355, 710–714. 28082413 10.1126/science.aai7919

[mpp12905-bib-0043] McLellan, H. , Boevink, P.C. , Armstrong, M.R. , Pritchard, L. , Gomez, S. , Morales, J. *et al* (2013) An RxLR effector from *Phytophthora infestans* prevents re‐localisation of two plant NAC transcription factors from the endoplasmic reticulum to the nucleus. PLoS Pathogens, 9, e1003670. 24130484 10.1371/journal.ppat.1003670 PMC3795001

[mpp12905-bib-0044] Oha, S. , Kim, H. and Choi, D. (2014) Rpi‐blb2‐mediated late blight resistance in *Nicotiana benthamiana* requires SGT1 and salicylic acid‐mediated signaling but not RAR1 or HSP90. FEBS Letters, 588, 1109–1115. 24582656 10.1016/j.febslet.2014.02.028

[mpp12905-bib-0045] Pawson, T. and Nash, P. (2003) Assembly of cell regulatory systems through protein interaction domains. Science, 300, 445–452. 12702867 10.1126/science.1083653

[mpp12905-bib-0046] Pieterse, C.M. , Van der Does, D.V. , Zamioudis, C. , Leon‐Reyes, A. and Van Wees, S.C. (2012) Hormonal modulation of plant immunity. Annual Review of Cell and Developmental Biology, 28, 489–521. 10.1146/annurev-cellbio-092910-154055 22559264

[mpp12905-bib-0047] Pitzschke, A. , Schikora, A. and Hirt, H. (2009) MAPK cascade signalling networks in plant defence. Current Opinion in Plant Biology, 12, 421–426. 19608449 10.1016/j.pbi.2009.06.008

[mpp12905-bib-0048] Qutob, D. , Kamoun, S. and Gijzen, M. (2002) Expression of a *Phytophthora sojae* necrosis‐inducing protein occurs during transition from biotrophy to necrotrophy. The Plant Journal, 32, 361–373. 12410814 10.1046/j.1365-313x.2002.01439.x

[mpp12905-bib-0049] Rehmany, A.P. , Gordon, A. , Rose, L.E. , Allen, R.L. , Armstrong, M.R. , Whisson, S.C. *et al* (2005) Differential recognition of highly divergent downy mildew avirulence gene alleles by RPP1 resistance genes from two *Arabidopsis* lines. The Plant Cell, 17, 1839–1850. 15894715 10.1105/tpc.105.031807 PMC1143081

[mpp12905-bib-0050] Shirasu, K. (2009) The HSP90‐SGT1 chaperone complex for NLR immune sensors. Annual Review of Plant Biology, 60, 139–164. 10.1146/annurev.arplant.59.032607.092906 19014346

[mpp12905-bib-0051] Sohn, K.H. , Lei, R. , Nemri, A. and Jones, J.D. (2007) The downy mildew effector proteins ATR1 and ATR13 promote disease susceptibility in *Arabidopsis thaliana* . The Plant Cell, 19, 4077–4090. 18165328 10.1105/tpc.107.054262 PMC2217653

[mpp12905-bib-0052] Tyler, B.M. (2007) *Phytophthora sojae*: root rot pathogen of soybean and model oomycete. Molecular Plant Pathology, 8, 1–8. 20507474 10.1111/j.1364-3703.2006.00373.x

[mpp12905-bib-0053] Tyler, B.M. , Tripathy, S. , Zhang, X. , Dehal, P. , Jiang, R.H. , Aerts, A. *et al* (2006) *Phytophthora* genome sequences uncover evolutionary origins and mechanisms of pathogenesis. Science, 313, 1261–1266. 16946064 10.1126/science.1128796

[mpp12905-bib-0054] Wang, H. , Ren, Y. , Zhou, J. , Du, J. , Hou, J. , Jiang, R. *et al* (2017) The cell death triggered by the nuclear localized RxLR effector PITG_22798 from *Phytophthora infestans* is suppressed by the effector AVR3b. International Journal of Molecular Sciences, 18, 409. 10.3390/ijms18020409 PMC5343943 28216607

[mpp12905-bib-0055] Wang, Q. , Han, C. , Ferreira, A.O. , Yu, X. , Ye, W. , Tripathy, S. *et al* (2011) Transcriptional programming and functional interactions within the *Phytophthora sojae* RXLR effector repertoire. The Plant Cell, 23, 2064–2086. 21653195 10.1105/tpc.111.086082 PMC3160037

[mpp12905-bib-0057] Wang, Y. and Wang, Y. (2018a) *Phytophthora sojae* effectors orchestrate warfare with host immunity. Current Opinion in Microbiology, 46, 7–13. 29454192 10.1016/j.mib.2018.01.008

[mpp12905-bib-0059] Wang, Y. , Xu, Y. , Sun, Y. , Wang, H. , Qi, J. , Wan, B. *et al* (2018b) Leucine‐rich repeat receptor‐like gene screen reveals that *Nicotiana* RXEG1 regulates glycoside hydrolase 12 MAMP detection. Nature Communications, 9, 594. 10.1038/s41467-018-03010-8 PMC5807360 29426870

[mpp12905-bib-0056] Wang, S. , McLellan, H. , Bukharova, T. , He, Q. , Murphy, F. , Shi, J. *et al* (2019a) *Phytophthora infestans* RXLR effectors act in concert at diverse subcellular locations to enhance host colonization. Journal of Experimental Botany, 70, 343–356. 30329083 10.1093/jxb/ery360 PMC6305197

[mpp12905-bib-0058] Wang, Y. , Tyler, B.M. and Wang, Y. (2019b) Defense and counterdefense during plant‐pathogenic oomycete infection. Annual Review of Microbiology, 73, 667–696. 10.1146/annurev-micro-020518-120022 31226025

[mpp12905-bib-0060] Wawra, S. , Belmonte, R. , Löbach, L. , Saraiva, M. , Willems, A. and van West, P. (2012) Secretion, delivery and function of oomycete effector proteins. Current Opinion in Microbiology, 15, 685–691. 23177095 10.1016/j.mib.2012.10.008

[mpp12905-bib-0061] Win, J. , Krasileva, K.V. , Kamoun, S. , Shirasu, K. , Staskawicz, B.J. and Banfield, M.J. (2012) Sequence divergent RXLR effectors share a structural fold conserved across plant pathogenic oomycete species. PLoS Pathogens, 8, e1002400. 22253591 10.1371/journal.ppat.1002400 PMC3257287

[mpp12905-bib-0062] Whisson, S.C. , Boevink, P.C. , Moleleki, L. , Avrova, A.O. , Morales, J.G. , Gilroy, E.M. *et al* (2007) A translocation signal for delivery of oomycete effector proteins into host plant cells. Nature, 450, 115. 17914356 10.1038/nature06203

[mpp12905-bib-0063] Xiang, J. , Li, X. , Yin, L. , Liu, Y. , Zhang, Y. , Qu, J. *et al* (2017) A candidate RxLR effector from *Plasmopara viticola* can elicit immune responses in *Nicotiana benthamiana* . BMC Plant Biology, 17, 75. 28410577 10.1186/s12870-017-1016-4 PMC5391559

[mpp12905-bib-0064] Xiang, J. , Li, X. , Wu, J. , Yin, L. , Zhang, Y. and Lu, J. (2016) Studying the mechanism of *Plasmopara viticola* RxLR effectors on suppressing plant immunity. Frontiers in Microbiology, 7, 709. 27242731 10.3389/fmicb.2016.00709 PMC4870276

[mpp12905-bib-0065] Xiong, Q. , Ye, W. , Choi, D. , Wong, J. , Qiao, Y. , Tao, K. *et al* (2014) *Phytophthora* suppressor of RNA silencing 2 is a conserved RxLR effector that promotes infection in soybean and *Arabidopsis thaliana* . Molecular Plant‐Microbe Interactions, 27, 1379–1389. 25387135 10.1094/MPMI-06-14-0190-R

[mpp12905-bib-0066] Yang, B. , Wang, Q. , Jing, M. , Guo, B. , Wu, J. , Wang, H. *et al* (2017) Distinct regions of the *Phytophthora* essential effector Avh238 determine its function in cell death activation and plant immunity suppression. New Phytologist, 214, 361–375. 28134441 10.1111/nph.14430

[mpp12905-bib-0067] Ye, W. , Wang, Y. , Shen, D. , Li, D. , Pu, T. , Jiang, Z. *et al* (2016) Sequencing of the litchi downy blight pathogen reveals it is a *Phytophthora* species with downy mildew‐like characteristics. Molecular Plant‐Microbe Interactions, 29, 573–583. 27183038 10.1094/MPMI-03-16-0056-R

[mpp12905-bib-0068] Yin, L. , An, Y. , Qu, J. , Li, X. , Zhang, Y. , Dry, I. *et al* (2017) Genome sequence of *Plasmopara viticola* and insight into the pathogenic mechanism. Scientific Reports, 7, 46553. 28417959 10.1038/srep46553 PMC5394536

[mpp12905-bib-0069] Yin, X. , Shang, B. , Dou, M. , Liu, R. , Chen, T. , Xiang, G. *et al* (2019) The nuclear‐localized RxLR effector PvAvh74 From *Plasmopara viticola* induces cell death and immunity responses in *Nicotiana benthamiana* . Frontiers in Microbiology, 10, 1531. 31354650 10.3389/fmicb.2019.01531 PMC6636413

[mpp12905-bib-0070] Yu, X. , Tang, J. , Wang, Q. , Ye, W. , Tao, K. , Duan, S. *et al* (2012) The RxLR effector Avh241 from *Phytophthora sojae* requires plasma membrane localization to induce plant cell death. New Phytologist, 196, 247–260. 22816601 10.1111/j.1469-8137.2012.04241.x

[mpp12905-bib-0071] Zhang, L. , Ni, H. , Du, X. , Wang, S. , Ma, X.W. , Nurnberger, T. *et al* (2017) The *Verticillium*‐specific protein VdSCP7 localizes to the plant nucleus and modulates immunity to fungal infections. New Phytologist, 215, 368–381. 28407259 10.1111/nph.14537

[mpp12905-bib-0072] Zheng, L. , Situ, J. , Zhu, Q. , Xi, P. , Zheng, Y. , Liu, H. *et al* (2019) Identification of volatile organic compounds for the biocontrol of postharvest litchi fruit pathogen *Peronophythora litchii* . Postharvest Biology and Technology, 155, 37–46.

