# Supplementary figures and images for: An RXLR effector PlAvh142 from Peronophythora litchii triggers plant cell death and contributes to virulence
Source: Mol Plant Pathol. 2020 Jan 7;21(3):415–28. doi: 10.1111/mpp.12905 (PMC7036370; doi:10.1111/mpp.12905)

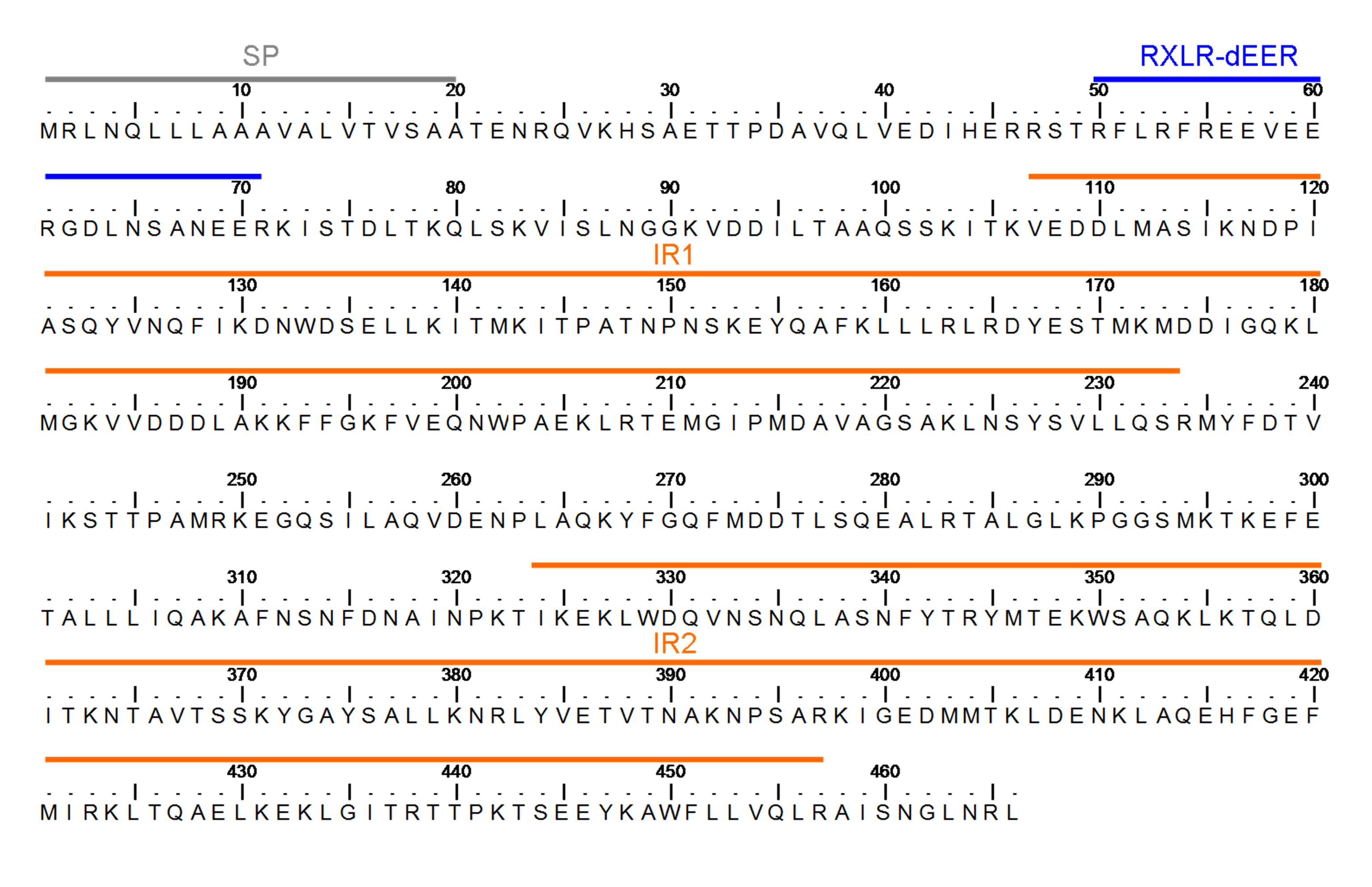

Supplement: Supplementary file 1 — FIGURE S1 Protein sequences of PlAvh142. Different regions are marked with bold lines [file MPP-21-415-s001.jpg]

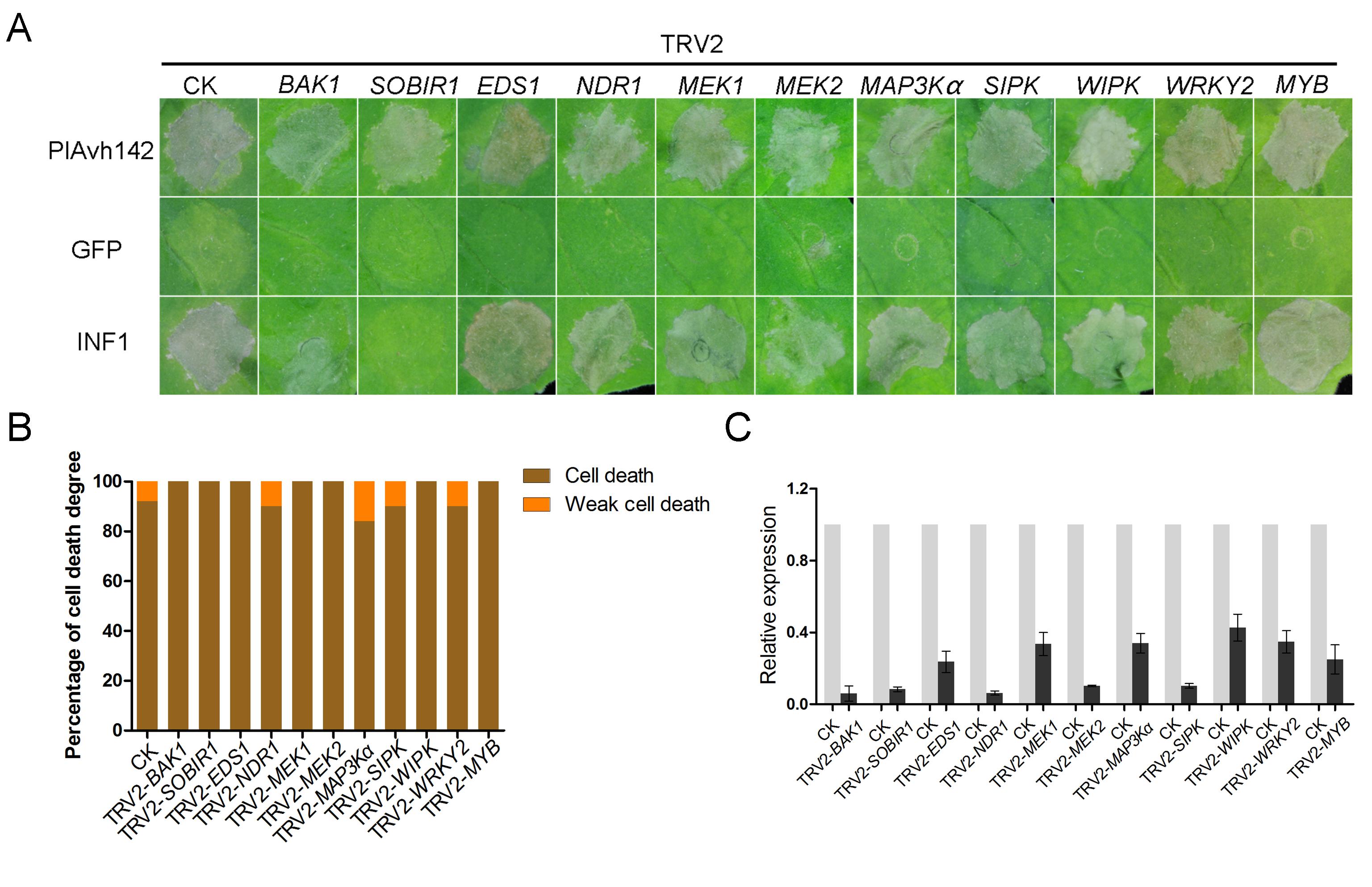

Supplement: Supplementary file 2 — FIGURE S2 Analysis of the components involved in PlAvh142‐induced cell death. Nicotiana benthamiana leaves were agroinfiltrated with pTRV2 constructs targeting BAK1, SOBIR1, EDS1, NDR1, MEK1, MEK2, MAP3Kα, SIPK, WIPK, WRKY2, and MYB; pTRV2::GFP was used as a control. (a) Representative images of PlAvh142‐induced cell death in silenced N. benthamiana leaves at 5 days post‐agroinfiltration (dpa). Agrobacterium tumefaciens carrying PlAvh142 was infiltrated into the upper leaves of silenced plants at 16–20 dpa of TRV constructs. (B) Quantification of cell death in N. benthamiana leaves scored at 5 dpa. The degree of cell death was divided into three levels: no cell death, weak cell death, and strong cell death. Asterisks indicate significant differences from green fluorescence protein (GFP)‐silenced plants (Wilcoxon rank‐sum test: ***, p < .001). (C) The transcript abundance of the genes in corresponding silenced plants was detected by RT‐qPCR. The constitutive expression gene NbEF1α was used as internal reference. Error bars represent the SD of three biological replicates. Similar results were obtained from three independent experiments [file MPP-21-415-s002.jpg]

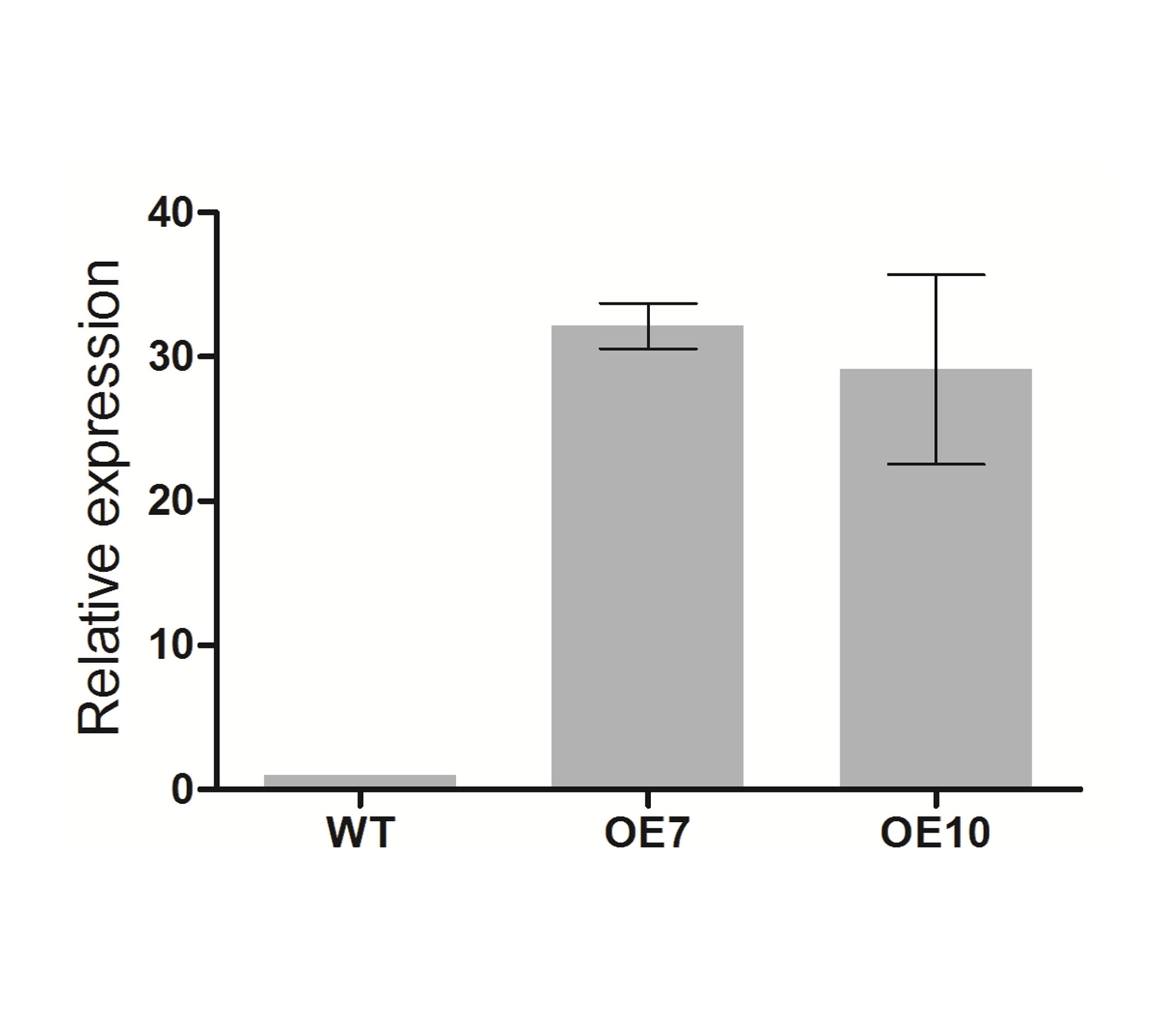

Supplement: Supplementary file 3 — FIGURE S3 The relative expression level of PlAvh142‐overexpressing mutants. RT‐qPCR was used to determine the overexpressing level of the mutants. The relative expression level was calibrated to the levels for the wild type that set as 1. The constitutive expression gene PlActin was used as internal reference. Error bars represent the SD of three biological replicates [file MPP-21-415-s003.jpg]

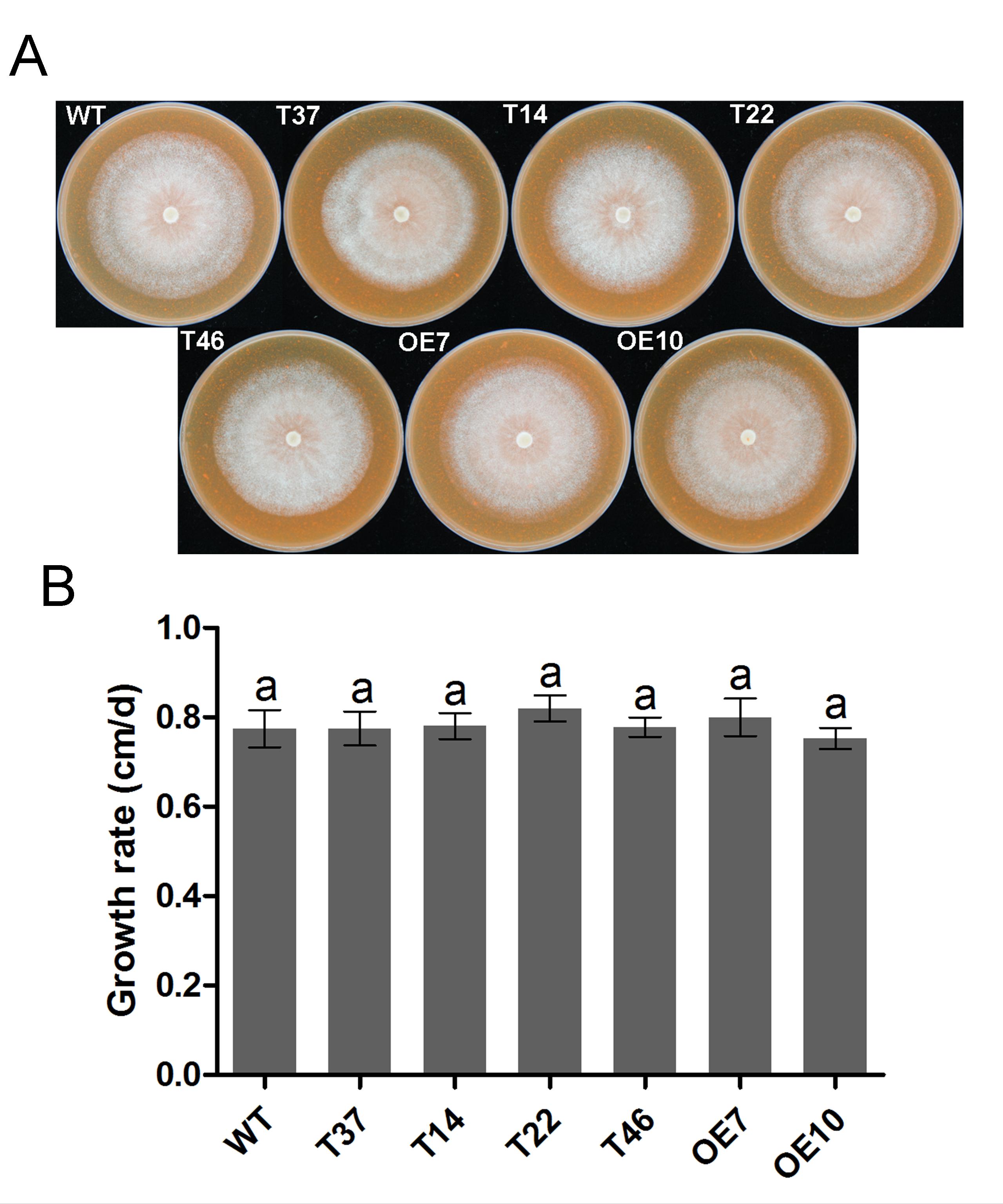

Supplement: Supplementary file 4 — FIGURE S4 The phenotype of PlAvh142 mutants were identical to wild type. (a) Colony morphology of PlAvh142 mutants grown on carrot juice agar medium after 5 days. (b) Growth rates of PlAvh142 mutants. Letters represent significant differences (p < .05; Duncan’s multiple range test). Similar results were obtained from three independent experiments [file MPP-21-415-s004.jpg]
